# TOP-LD: A tool to explore linkage disequilibrium with TOPMed whole-genome sequence data

**DOI:** 10.1016/j.ajhg.2022.04.006

**Published:** 2022-05-02

**Authors:** Le Huang, Jonathan D. Rosen, Quan Sun, Jiawen Chen, Marsha M. Wheeler, Ying Zhou, Yuan-I Min, Charles Kooperberg, Matthew P. Conomos, Adrienne M. Stilp, Stephen S. Rich, Jerome I. Rotter, Ani Manichaikul, Ruth J.F. Loos, Eimear E. Kenny, Thomas W. Blackwell, Albert V. Smith, Goo Jun, Fritz J. Sedlazeck, Ginger Metcalf, Eric Boerwinkle, NHLBI Trans-Omics for Precision Medicine (TOPMed) Consortium, Laura M. Raffield, Alex P. Reiner, Paul L. Auer, Yun Li

**Affiliations:** 1 Curriculum in Bioinformatics and Computational Biology, University of North Carolina at Chapel Hill, Chapel Hill, NC 27599, USA; 2 Department of Biostatistics, University of North Carolina at Chapel Hill, Chapel Hill, NC 27599, USA; 3 Department of Genome Sciences, University of Washington, Seattle, WA 98105, USA; 4 Division of Public Health Sciences, Fred Hutchinson Cancer Research Center, Seattle, WA 98109, USA; 5 Department of Medicine, University of Mississippi Medical Center, Jackson, MS 39216, USA; 6 Department of Biostatistics, University of Washington, Seattle, WA 98105, USA; 7 Center for Public Health Genomics, Department of Public Health Sciences, University of Virginia School of Medicine, Charlottesville, VA 22908, USA; 8 The Institute for Translational Genomics and Population Sciences, Department of Pediatrics, The Lundquist Institute for Biomedical Innovation at Harbor-UCLA Medical Center, Torrance, CA 90502, USA; 9 The Charles Bronfman Institute for Personalized Medicine, Icahn School of Medicine at Mount Sinai, New York City, NY 10029, USA; 10 Novo Nordisk Foundation Center for Basic Metabolic Research, Faculty of Health and Medical Sciences, University of Copenhagen, 2200 Copenhagen, Denmark; 11 TOPMed Informatics Research Center, University of Michigan, Department of Biostatistics, Ann Arbor, MI 48109, USA; 12 Human Genetics Center, School of Public Health, The University of Texas Health Science Center at Houston, Houston, TX 77030, USA; 13 Human Genome Sequencing Center, Baylor College of Medicine, Houston, TX 77030, USA; 14 Human Genetics Center, Department of Epidemiology, Human Genetics, and Environmental Sciences, School of Public Health, The University of Texas Health Science Center at Houston, Houston, TX 77030, USA; 15 Department of Genetics, University of North Carolina at Chapel Hill, Chapel Hill, NC 27599, USA; 16 Department of Epidemiology, University of Washington, Seattle, WA 98195, USA; 17 Division of Biostatistics, Institute for Health and Equity, and Cancer Center, Medical College of Wisconsin, Milwaukee, WI 53226, USA; 18 Department of Computer Science, University of North Carolina at Chapel Hill, Chapel Hill, NC 27599, USA

## Abstract

Current publicly available tools that allow rapid exploration of linkage disequilibrium (LD) between markers (e.g., HaploReg and LDlink) are based on whole-genome sequence (WGS) data from 2,504 individuals in the 1000 Genomes Project. Here, we present TOP-LD, an online tool to explore LD inferred with high-coverage (∼30×) WGS data from 15,578 individuals in the NHLBI Trans-Omics for Precision Medicine (TOPMed) program. TOP-LD provides a significant upgrade compared to current LD tools, as the TOPMed WGS data provide a more comprehensive representation of genetic variation than the 1000 Genomes data, particularly for rare variants and in the specific populations that we analyzed. For example, TOP-LD encompasses LD information for 150.3, 62.2, and 36.7 million variants for European, African, and East Asian ancestral samples, respectively, offering 2.6- to 9.1-fold increase in variant coverage compared to HaploReg 4.0 or LDlink. In addition, TOP-LD includes tens of thousands of structural variants (SVs). We demonstrate the value of TOP-LD in fine-mapping at the *GGT1* locus associated with gamma glutamyltransferase in the African ancestry participants in UK Biobank. Beyond fine-mapping, TOP-LD can facilitate a wide range of applications that are based on summary statistics and estimates of LD. TOP-LD is freely available online.

## Main text

Linkage disequilibrium (LD), i.e., the non-random association of alleles at different variant sites in a given population, is an important genetic phenomenon. Patterns of LD between genetic markers can be leveraged to gain insights in a variety of different applications, from population genetic research to disease association studies.^1^^,^^2^ With the growth of whole-genome sequencing (WGS) and high-throughput array and genotype imputation technologies, resources for calculating LD across populations have expanded to encompass multiple populations at variant sites with increasingly rare frequencies.^3^, ^4^, ^5^, ^6^ Due to the centrality of LD in a host of applications, multiple tools exist for querying LD between genetic markers in different populations. The current most widely used LD lookup tools, HaploReg^7^ and LDlink,^8^ base their LD estimates on the 1000 Genomes data. Specifically, HaploReg uses phase 1 and LDlink uses phase 3 1000 Genomes data. Although the 1000 Genomes data contains LD information on >99% of genetic markers with minor allele frequency (MAF) > 1% in a variety of populations,^4^ there remains a dearth of publicly available information on LD between markers with MAF < 1%. We have created a new LD lookup tool (called “TOP-LD”), in the spirit of HaploReg and LDlink, that is based on deep (30×) WGS data from the NHLBI Trans-Omics for Precision Medicine (TOPMed) Program. Because the TOPMed data contain much larger sample sizes with greater depth of sequencing than the 1000 Genomes project, TOP-LD provides a significant upgrade in LD information availability, specifically by including single-nucleotide variants and small indels (referred to hereafter simply as “SNVs”) with MAF < 1% as well as structural variants (SVs). Here, we describe the data and methods that went into creating TOP-LD along with specific examples of how TOP-LD can provide essential information that is missed by HaploReg and LDlink.

**Figure 1:**
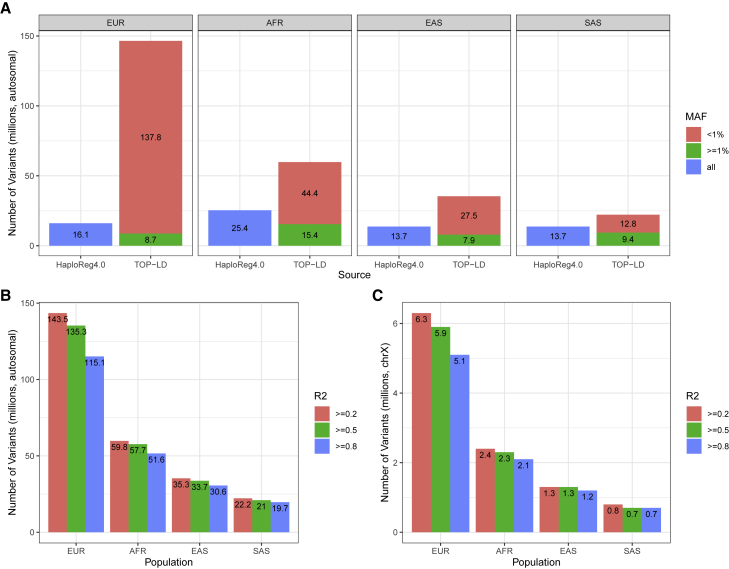
Number of variants included in TOP-LD (A) Comparison of autosomal variants with HaploReg 4.0 by population. Blue bars on the left show total number of autosomal variants in HaploReg4.0. Green and red indicate common (MAF ≥ 1%) and uncommon (MAF < 1%) autosomal variants in TOP-LD. Note that HaploReg4.0 provides LD for ASN (Asian) with no separate information for EAS and SAS. Therefore, we used the same 13.7 million ASN variants for comparison in both EAS and SAS. (B) Number of autosomal variants in TOP-LD breaking down by LD R^2^ threshold. The majority of the variants have at least one LD proxy with R^2^ ≥ 0.8. (C) Number of chrX variants in TOP-LD breaking down by LD R^2^ threshold. (Note: LD information downloaded from HaploReg4.0 does not contain chromosome X. Therefore, we compared TOP-LD with HaploReg4.0 only for autosomal variants).

We used TOPMed WGS data^6^ from the following four cohorts: BioMe Biobank (BioMe), the Multi-Ethnic Study of Atherosclerosis (MESA), the Jackson Heart Study (JHS), and the Women’s Health Initiative (WHI). We aimed to provide LD estimates for genetically homogeneous groups of individuals from one of the following four ancestral populations: European (EUR), African (AFR), East Asian (EAS), and South Asian (SAS). To select appropriate samples, we first inferred local and global ancestry for all participants in these four cohorts by using RFMix,^9^ with reference populations including five ancestral groups, namely African, Native American, East Asian, European, and South Asian. After local ancestry inference, we then retained only TOPMed samples with >90% estimated ancestry from a single population, as estimated via RFMix. We further removed related individuals by using a stringent kinship coefficient threshold of 2^−5.5^ obtained via PC-Relate.^10^ This threshold of 2^−5.5^ removes pairs within as far as fifth degree relationship. The final dataset included 1,335 unrelated individuals of African, 844 of East Asian, 13,160 of European, and 239 of South Asian ancestry for pairwise LD inference. Regarding variants, we started with all TOPMed freeze 8 polymorphic variants that passed quality control and retained multi-allelic variants or multiple entries at the same position, resulting in a total of 23.0–153.0 million SNVs in each of the ancestral groups (Figure 1A, Table S1).

We inferred LD separately within each of the four ancestral groups, for all pairs of variants within 1 Mb of each other, and retained LD pairs meeting a minimum R^2^ threshold of 0.2. The reported R^2^ between two variants is the squared Pearson correlation coefficient between their phased haplotypes, where phasing was performed with Eagle 2.4 for all polymorphic variants, similar to phasing of the freeze 5 data.^6^ No minimum minor allele count thresholding was used, that is, even singletons in our sample were included in LD calculations. We also report the direction of each association as either positive (+) or negative (−) on the basis of the sign of the Pearson correlation coefficient between the corresponding pair of reference (REF) alleles. In addition to R^2^, we also report D-prime statistics for each pair of variants meeting the R^2^ of 0.2.

We filtered chromosome X to exclude the pseudo-autosomal regions: PAR1 (bp 10,001–2,781,479, GRCh38) and PAR2 (bp 155,701,383–156,030,895, GRCh38). Variants that were not coded as homozygous in the males were excluded from the LD calculations. We inferred LD for the remaining variants by using a total of 2F + M haplotypes, where F and M are the numbers of females and males, respectively.

The TOPMed structural variant (SV) call-set freeze 1 was merged with a reduced TOPMed SNV call-set where SNVs with MAF < 0.1% were filtered out before merging, and then the merged SV-SNV dataset was phased with Eagle2.^11^ SVs with >10% missingness were removed prior to phasing. For each ancestry group, we included 16.5–79K SVs (deletions, duplications, and inversion) with the majority being lower frequency (e.g., 7–69K with MAF < 1%) (Table 1). LD values were subsequently estimated as the squared Pearson correlation coefficient between the corresponding pair of phased alleles.

**Table 1:** Summary of SVs by population

Population	Number of SVs	Number of SVs in LD w/SNVs^a^	Number of SVs with MAF < 0.01
EUR	79,004	16,301	69,011
AFR	44,859	15,151	27,978
SAS	16,511	10,392	7,292
EAS	20,789	7,498	12,902

a Number of SVs having at least one SNV LD tag with R^2^ ≥ 0.8.

**Figure 2:**
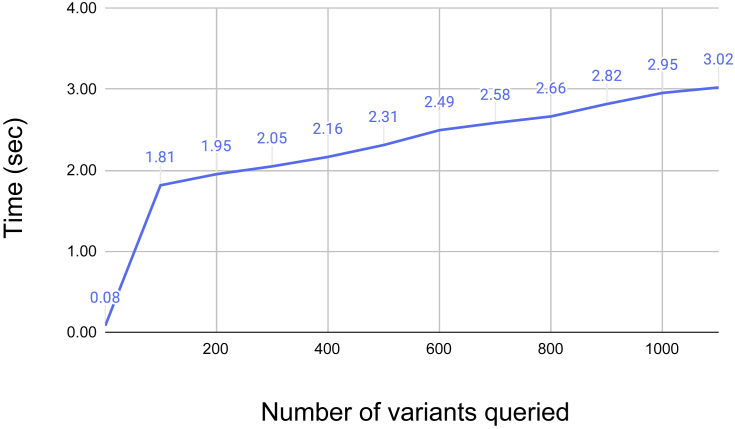
Elapsed time (in seconds) for queries The x axis represents the number of variants queried, and the y axis represents the elapsed time.

TOPMed LD information was then loaded into the TOP-LD website, which is powered by a combination of MySQL, PHP, Javascript, and Apache2 under the CloudSQL and Compute Engine of Google Cloud Platform. The web interface provides access to all precomputed LD estimates. Users have the option to either paste or upload a file containing variant(s) of interest. Users can specify the population (East Asian, European, African, or South Asian) in which LD was estimated. In TOP-LD, markers are identified by rsID, or chr:position, or chr:position:REF:ALT for SNVs, or TOPMed variant names for SVs (in the format of DEL/DUP/INV_chr:startPosition-endPosition, for example, DEL_10:85001–97300). TOP-LD returns all variants within a pre-specified LD threshold (ranging from R^2^ values of 0.2 to 1.0) with the query variant. TOP-LD supports fast batch queries (Figure 2); querying a single variant takes ∼0.5 s, while a batch query of 500 variants takes ∼2.3 seconds. TOP-LD currently allows a maximum of 500 variants in one query.

**Figure 3:**
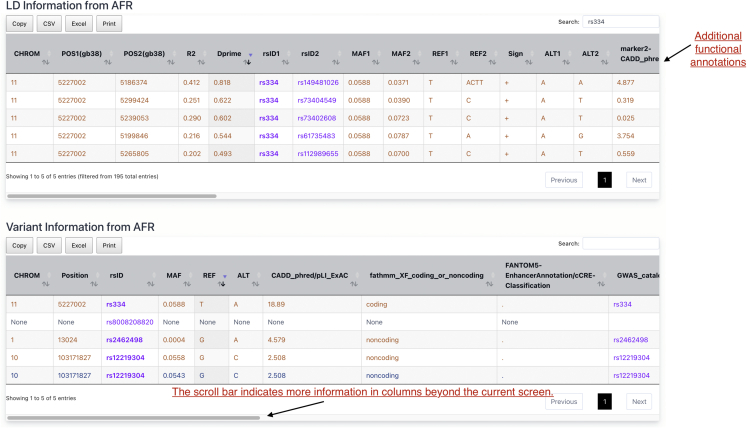
An example query result The result contains two parts. The top part “LD information from AFR” shows the LD information where each line provides information between a query variant (rsID1) and one of its corresponding LD proxies (rsID2). The bottom part “variant information from AFR” provides variant information, which shows basic information for each query variant. From the bottom part, we know that the user’s query includes four variants: rs334, rs8008208820, rs2462498, and rs12219304. Variants not included in LD calculation will have “none” records. For instance, rs8008208820 in this example query is not involved in LD inference and therefore will not have any LD proxies in the top part simply because of no data. Records from SV inference are in blue and those from SNV data are in orange. Some variants may appear twice because they are included in both SNV LD calculation and SV calculation. For example, in this example, rs12219304 appeared twice with MAF 0.0558 from the SNV source (second last record in orange) and MAF 0.0543 from the SV source (last record in blue).

After submitting the query, the website auto-directs to a result page that contains two parts: LD information on the top panel and variant information on the bottom panel. The latter provides basic information for the queried variants, including position, marker name, alleles (REF and ALT), and minor allele frequency (MAF). Markers not in the database will have “none” for all fields except marker names. The LD panel displays related LD metrics, one pair of variants on each line, including both R^2^, D′, and the sign of LD (measured between REF alleles of the two variants), along with marker name, marker position, alleles, and frequency for both variants in the pair (Figure 3). In addition, we provide the following pieces of information for SNVs from WGSA annotation^12^: CADD score (phred-scaled), fathmm_XF_coding_or_noncoding classification, FANTOM5 enhancer annotations, gene name, and relative location to gene as well as a link to GWAS catalog query results.^13^ For SVs, we provide a variety of annotations including gene(s) overlapping the SV, the SV’s location relative to gene, the gene’s pLI score, overlapping candidate *cis*-regulatory regions (cCREs) from ENCODE SCREEN.^14^^,^^15^ The query results can be sorted, searched, copied, exported, and printed for further analyses.

The TOP-LD tool leverages TOPMed WGS data, whose much larger sample size and high depth sequencing lead to LD information for a much larger number of variants compared to the 1000 Genomes Project. As shown in Figure 1A and Table S1, TOP-LD offers 2.6- to 9.1-fold increase in variant coverage compared to the other state-of-the-art resources such as HaploReg 4.0 or LDlink. For example, for the European population, TOP-LD includes 146.5 million autosomal SNVs, while HaploReg 4.0 or LDlink contains 16.1 million variants. Not surprisingly, the vast majority of the variants in TOP-LD that are not in 1000 Genomes, contributing to the up to 9.1× increase, are low frequency or rare. For example, out of the 146.5 million autosomal SNVs cataloged in the TOP-LD European population, 137.8 million have MAF < 0.01 (Figure 1A, Table S1). Most of the variants have LD proxies. For example, 115.1 out of the 146.5 (78.6%) million autosomal variants have at least one LD tag with R^2^ ≥ 0.8 and if we further relax the R^2^ threshold to 0.5 and 0.2, the number increases to 135.3 (92.4%) and 143.5 (98.0%), respectively (Figure 1B).

For chromosome X, we have included 6.5 million, 2.4 million, 1.3 million, and 760,000 variants for the European, African, East Asian, and South Asian populations, respectively (Table S1). Similar to the autosomal variants, the majority of these variants have at least one LD proxy with R^2^ ≥ 0.8: 5.1 million, European; 2.1 million, African; 1.2 million East Asian; 690,000, South Asian (Figure 1C, Table S2).

To evaluate the consistency between TOP-LD estimates and those from Haploreg v4.1, we collected the set of overlapping variants based on rsID with MAF ≥ 0.05 for Europeans and Africans. This set of variants was further filtered such that the MAF values were within 10% of each other because large MAF differences would induce large LD differences. Figures S1 and S2 show high level of agreement between TOP-LD and Haploreg v4.1 LD estimates (e.g., Pearson correlation = 0.972 and 0.962 for European and African chromosome 1, respectively). Similarly, comparison of the chromosome X TOP-LD estimates for females and males again show high level of consistency (Pearson correlation = 0.992 and 0.975 for European and African population, respectively) (Figures S3 and S4).

To demonstrate the utility of TOP-LD, we performed fine-mapping at the *GGT1* locus on chromosome 22, which is known to be associated with gamma glutamyltransferase.^16^ We performed sequential conditional analysis with EPACTS^17^ by using individual-level data among 8,768 UK Biobank participants of African ancestry following the same strategy in our previous work^18^ adjusting for the same covariates as in Sun et al.^19^ The sequential conditional analyses with individual-level data identified seven distinct signals at the *GGT1* locus associated with gamma glutamyltransferase (Table 2). Because we used individual-level data for this conditional analysis, we considered these seven distinct signals to be the “working truth.”

**Table 2:** Summary statistics of distinct working truth at *GGT1* locus associated with gamma glutamyltransferase

Signal	Variant	Position (hg38)	Effect allele	Unconditional p value	p value conditional on previous signals^a^	Effect allele frequency
1	rs4049904	24609759	G	2.82e−61	N/A	10.27%
2	rs73404962	24598530	G	4.46e−29	2.00e−36	5.63%
3	rs743369	24588099	A	9.94e−36	7.51e−27	11.94%
4	rs6004193	24598329	C	4.23e−41	3.25e−19	18.27%
5	rs57719575	24609020	C	3.97e−38	1.98e−24	14.86%
6	rs3876101	24607291	A	2.66e−15	1.17e−13	35.45%
7	rs116161010	24585912	T	5.69e−17	7.70e−9	7.13%

a The p values are reported from the sequential conditional analysis. For example, we report the p value for rs73404962 conditional on rs4049904, the p value of rs743369 conditional on both rs4049904 and rs73404962, and so forth.

We then carried out fine-mapping analysis with the FINEMAP method^20^ by using only GWAS summary statistics from Sun et al.^19^ We applied FINEMAP with an LD reference either from TOP-LD or from the 1000 Genomes Project and assessed the performance by comparing the results with “working truth” established from the sequential conditional analysis of the individual-level data.

FINEMAP produced 95% credible sets containing five variants when using either the 1000 Genomes (1000G) Project LD panel or the TOP-LD panel (see Table 3). However, the 1000G-based credible set contained only one of the seven signals from the “working truth” set. In contrast, the TOP-LD-based credible set contained three of the seven signals from the “working truth” set. In addition, because the lead variant from each conditional analysis (corresponding to each distinct signal) is selected somewhat arbitrarily, we also considered their LD proxies. When we considered any LD proxy (using a lenient R^2^ threshold of 0.2) of a variant in the working truth set, the 1000G-based results still only identified a single signal from the working truth, whereas the TOP-LD-based results identified four of the seven signals (Table 3).

**Table 3:** FINEMAP credible-set variants

		Variant 1	Variant 2	Variant 3	Variant 4	Variant 5
1000G reference	credible-set variant	rs4049904	rs147866692	rs570263050	rs115231893	22:24649848:G:A (hg38)
	LD with working truth	1 (w/rs4049904 itself)	0.464 (w/rs4049904)	0.606 (w/rs4049904)	0.275 (w/rs4049904)	0.434 (w/rs4049904)
TOP-LD reference	credible-set variant	rs4049904	rs743369	rs57719575	rs2073397	rs5751902
	LD with working truth	1 (w/rs4049904 itself)	1 (w/rs743369 itself)	1 (w/rs57719575 itself)	0.83 (w/rs6004193)	0.51 (w/rs6004193)

The two five-variant credible sets provided by FINEMAP with either 1000G or TOP-LD as reference. For each credible-set variant, we list the corresponding variant (and the LD Rsq) from the working truth that has the highest LD.

We also used TOP-LD to aid in the identification and prioritization of potentially causal structural variants at GWAS loci. For example, our recent association analysis^21^ with TOPMed data identified an African-specific (MAF = 0.129) variant rs28450540 associated with lower monocyte count (p = 3.65 × 10^−17^). Query for LD tags via TOP-LD revealed a ∼600 bp deletion near *S1PR3* in perfect LD (R^2^ = 1) with rs28450540 in the African population. We performed genome editing in monocytic and primary human HSPCs followed by xenotransplantation, which provides evidence that the deletion disrupts an *S1PR3* monocyte enhancer leading to decreased *S1PR3* expression. These preliminary data from functional experiments suggest that the 600 bp deletion is most likely casual but would have been missed in standard association analysis with only SNVs.^22^ TOP-LD offers a simple and efficient approach to rescue such putative causal structural variants.

LD information, reflecting recombination, natural selection, and demographic history, has always been of intense interest in population genetics and complex trait association studies. LD information is also indispensable for a wide range of other applications, including GWAS follow-up and many summary-statistics-based inferences including fine-mapping, imputation of association summary statistics, construction of polygenic risk scores (PRSs), and interpretation and prioritization of GWAS results for further functional and clinical studies. TOP-LD significantly boosts the coverage of lower frequency variants by harnessing the power of high-coverage (∼30×) WGS data of over 15,000 individuals primarily of a single continental ancestry. We demonstrate the utility of TOP-LD in fine-mapping at the *GGT1* locus and variant prioritization at the *S1PR3* locus. The LD information provided by TOP-LD will facilitate a range of essential inferences for common and rare variation across a diverse range of populations.

## Data Availability

Data generated for this study can be accessed via the TOP-LD web portal: http://topld.genetics.unc.edu.

## Web resources

EPACTS, https://genome.sph.umich.edu/wiki/EPACTS

FINEMAP, http://www.christianbenner.com/

HaploReg, https://pubs.broadinstitute.org/mammals/haploreg/

LDlink, https://ldlink.nci.nih.gov/

TOP-LD, http://topld.genetics.unc.edu/

TOPMed, https://topmed.nhlbi.nih.gov/

## Supplemental information

Document S1. Supplemental methods, Figures S1–S4, and Tables S1 and S2

Document S2. Article plus supplemental information
