# Supplementary material for: TOP-LD: A tool to explore linkage disequilibrium with TOPMed whole-genome sequence data
Source: Am J Hum Genet. 2022 May 2;109(6):1175–81. doi: 10.1016/j.ajhg.2022.04.006 (PMC9247832; doi:10.1016/j.ajhg.2022.04.006)
Supplement: Document S1. Supplemental methods, Figures S1–S4, and Tables S1 and S2 [file mmc1.pdf]

The American Journal of Human Genetics, Volume *109*

Supplemental information

TOP-LD: A tool to explore linkage disequilibrium with TOPMed whole-genome sequence data

Le Huang, Jonathan D. Rosen, Quan Sun, Jiawen Chen, Marsha M. Wheeler, Ying Zhou, Yuan-I Min, Charles Kooperberg, Matthew P. Conomos, Adrienne M. Stilp, Stephen S. Rich, Jerome I. Rotter, Ani Manichaikul, Ruth J.F. Loos, Eimear E. Kenny, Thomas W. Blackwell, Albert V. Smith, Goo Jun, Fritz J. Sedlazeck, Ginger Metcalf, Eric Boerwinkle, NHLBI Trans-Omics for Precision Medicine (TOPMed) Consortium, Laura M. Raffield, Alex P. Reiner, Paul L. Auer, and Yun Li

Supplementary Figures

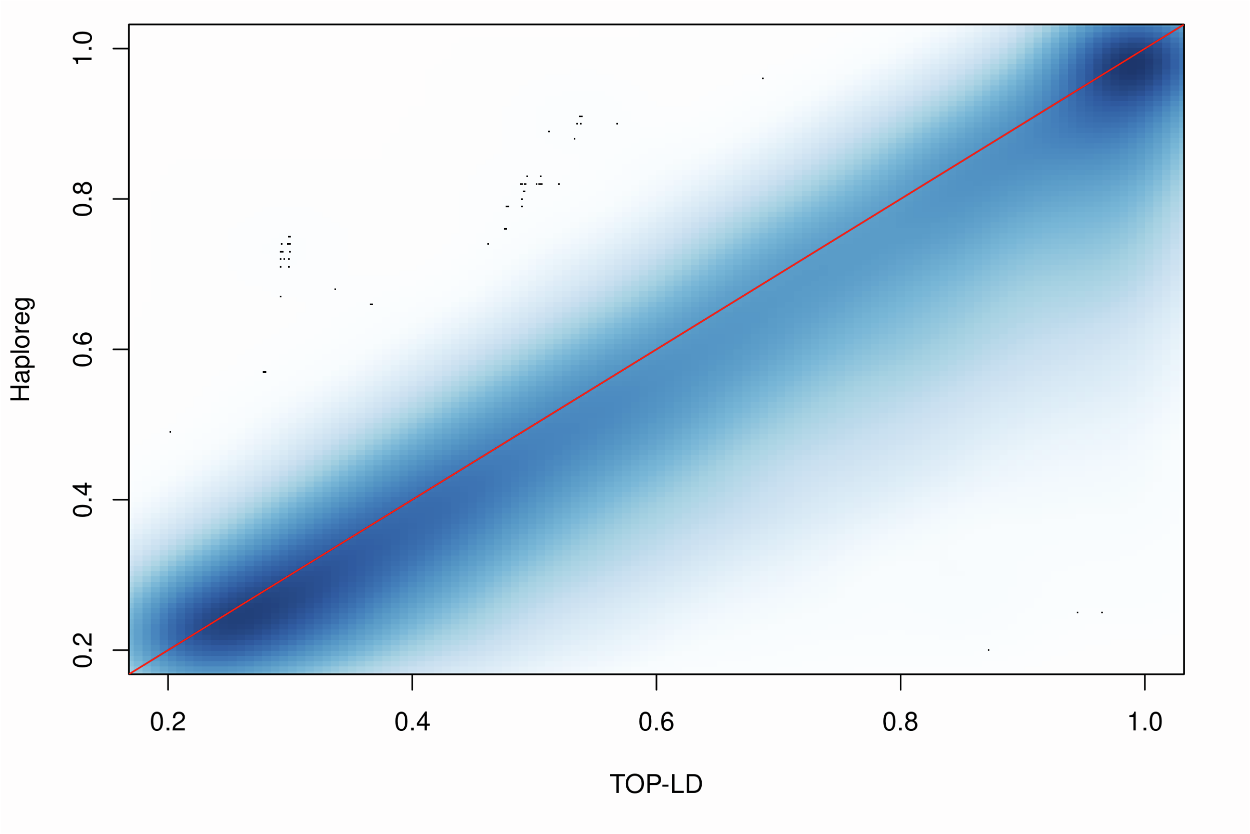

Figure S1: Smooth scatter plot of LD R-squared values from TOP-LD (x-axis) and Haploreg (y-axis) for pairs of variants with MAF>5% on chromosome 1 in European populations.

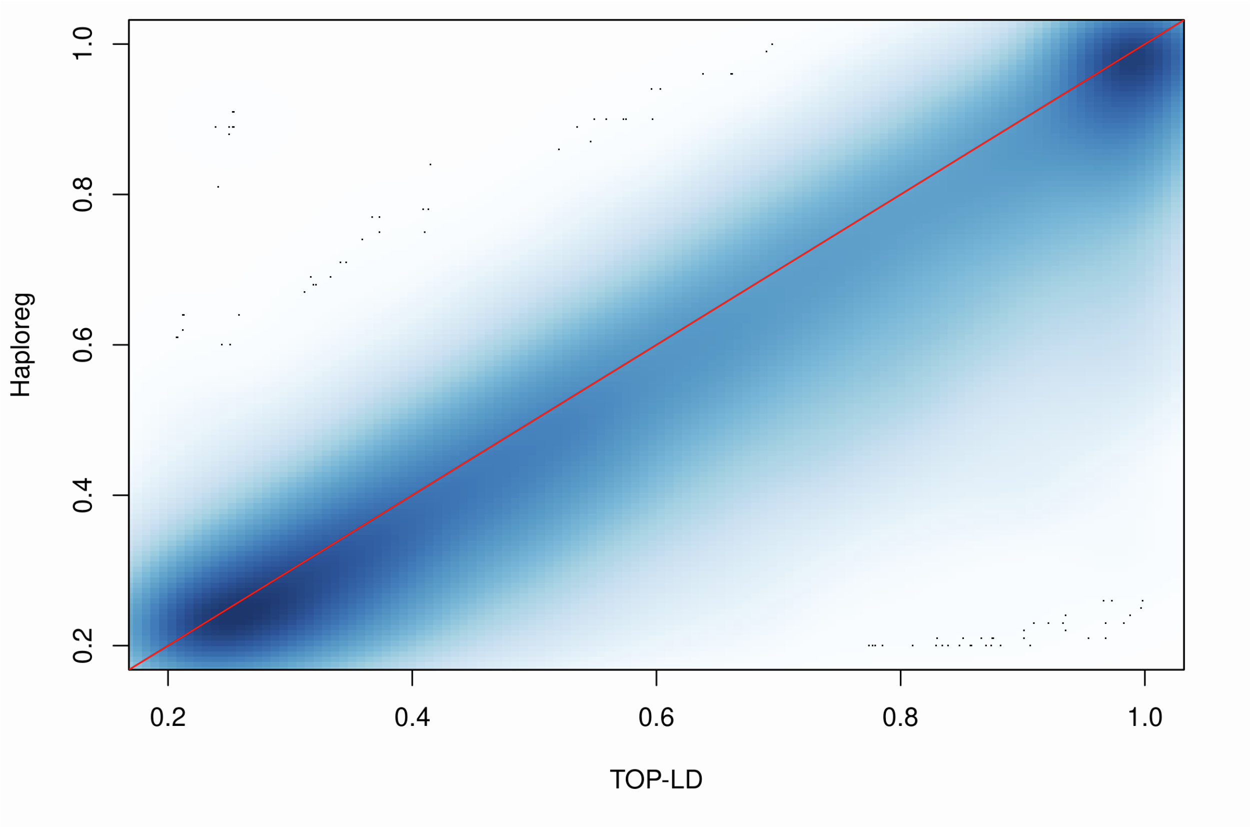

Figure S2: Smooth scatter plot of LD R-squared values from TOP-LD (x-axis) and Haploreg (y-axis) for pairs of variants with MAF>5% on chromosome 1 in African populations.

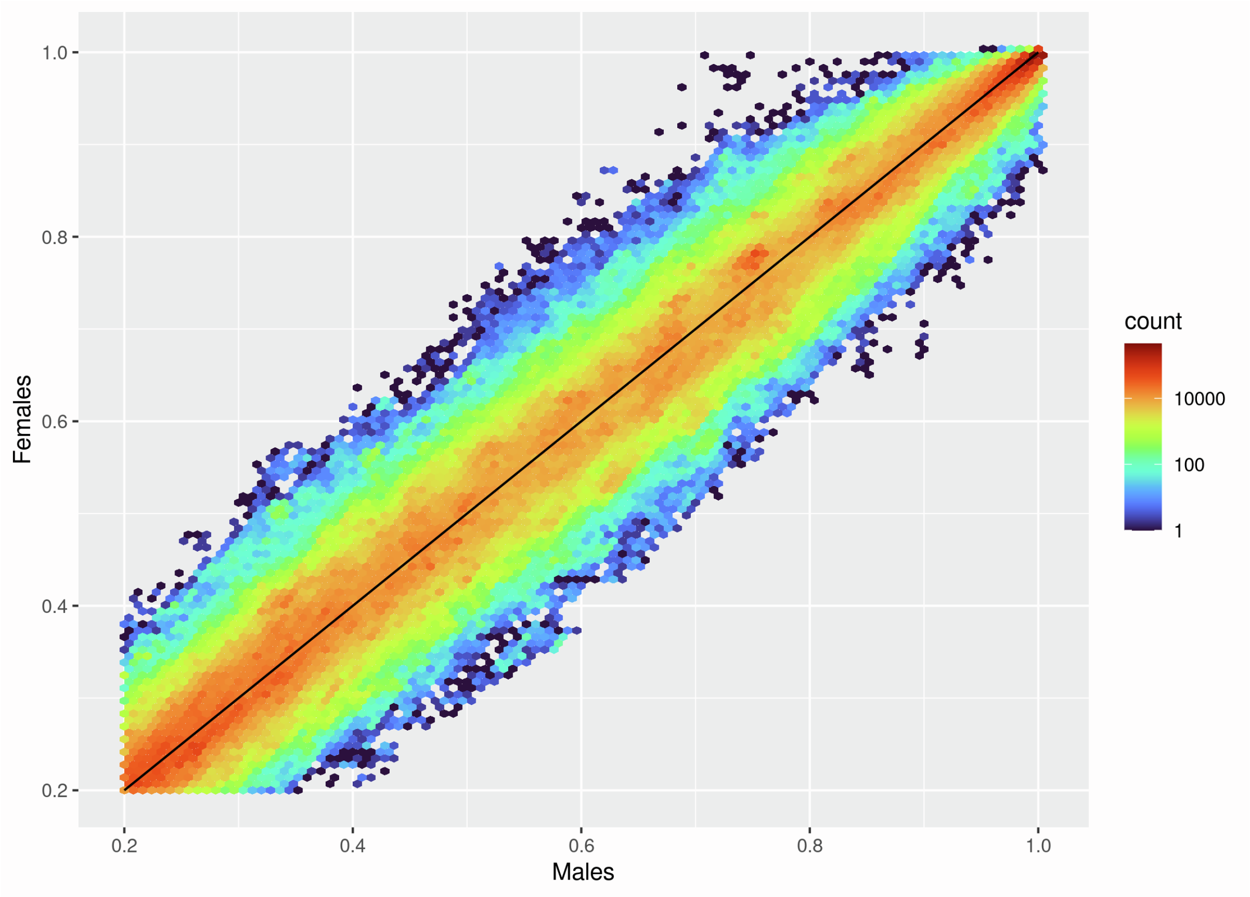

Figure S3: Hexbin plot of LD R-squared values between males (x-axis) and females (y-axis) between pairs of variants with MAF>5%, not in PAR1 or PAR2, on chromosome X in European populations.

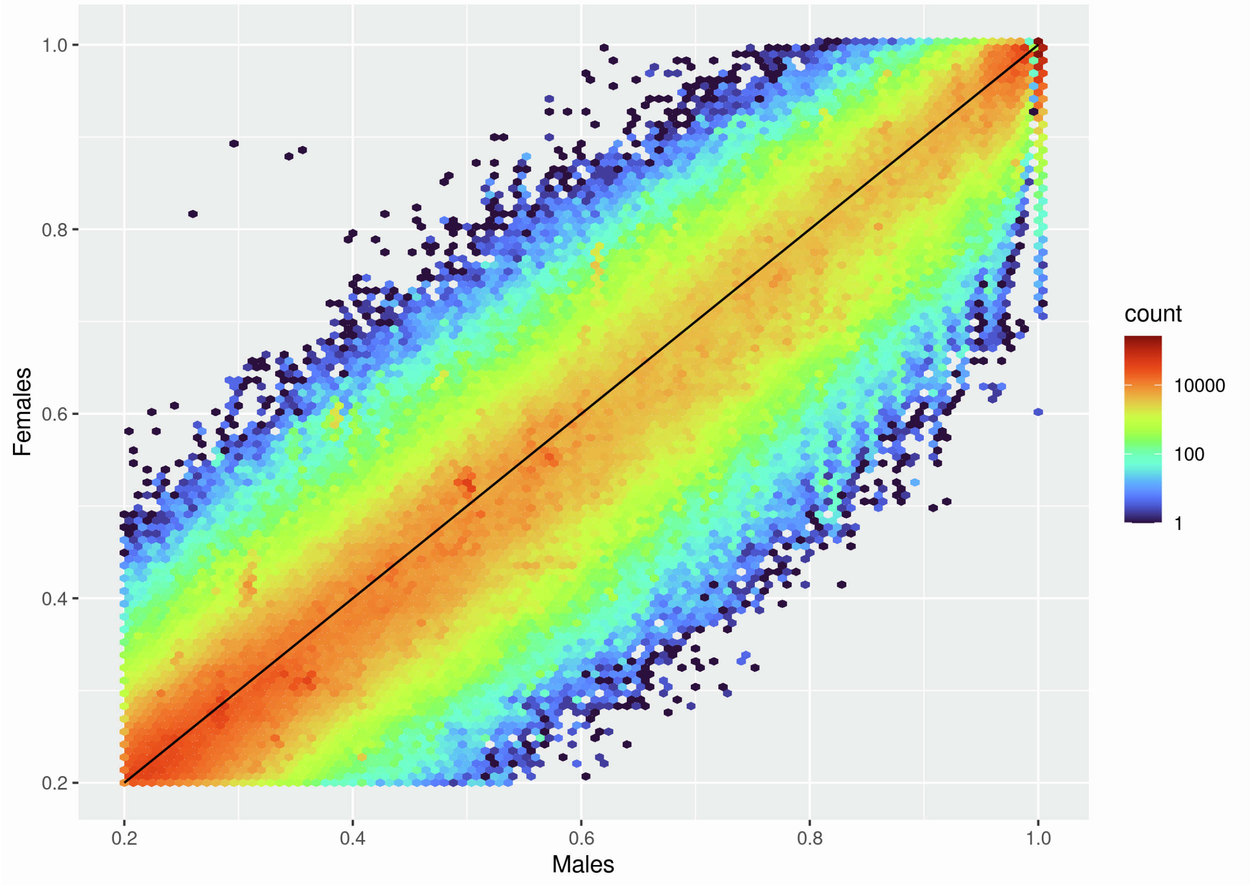

Figure S4: Hexbin plot of LD R-squared values between males (x-axis) and females (y-axis) between pairs of variants with MAF>5%, not in PAR1 or PAR2, on chromosome X in African populations.

Supplementary Tables

Table S1: Summary of SNVs and small indels by population by MAF.

Population	#TOP-LD variants[a] (MAF >0) in millions (chrX[b])	#TOP-LD variants[a] (MAF <1%) in millions (chrX[b])	#autosomal[c] variants in HaploReg4.0 in millions
EUR	153.0 (6.5)	144.0 (6.2)	16.1
AFR	62.2 (2.4)	46.2 (1.8)	25.4
SAS	23.0 (0.8)	13.3 (0.5)	13.7[d]
EAS	36.7 (1.3)	28.6 (1.1)	

a: number of unique variants, genome-wide (including autosomes and chromosome X)
b: number of unique variants on chromosome X
c: based on HaploReg LD information downloaded from https://pubs.broadinstitute.org/mammals/haploreg/data/, which does not contain chromosome X.
d: HaploReg4.0 provides LD for ASN (Asian), with no separate information for SAS and EAS.

Table S2. Summary of SNVs and small indels by population by varying LD R^2 thresholds

Population	#variants[a] ($R^2 \geq 0.2$), in millions (chrX[b])	#variants[a] ($R^2 \geq 0.5$), in millions (chrX[b])	#variants[a] ($R^2 \geq 0.8$) in millions (chrX[b])
EUR	149.8 (6.3)	141.2 (5.9)	120.2 (5.1)
AFR	62.2 (2.4)	60.0 (2.3)	53.7 (2.1)
SAS	23.0 (0.8)	21.7 (0.7)	20.4 (0.7)
EAS	36.6 (1.3)	35.0 (1.3)	31.8 (1.2)

a: number of unique variants, genome-wide (including autosomes and chromosome X) from LD pairs with $R^2 \geq$ a certain threshold
b: number of unique variants on chromosome X

Supplementary Methods

TOPMed samples

NHLBI's TOPMed program is comprised of many parent studies, including four ancestrally diverse studies that contributed to our analyses including BioMe Biobank (BioMe)[1], Jackson Heart Study (JHS)[2, 3], Multi-Ethnic Study of Atherosclerosis (MESA)[4], and Women's Health Initiative (WHI) [5]. Additional information about the design of each study and the sampling of individuals within each cohort for WGS is available in the *Cohort Descriptions* section below. All studies were approved by the appropriate institutional review boards (IRBs), and informed consent was obtained from all participants.

TOPMed whole genome sequencing and quality control

WGS was performed at an average depth of 38X by six sequencing centers (Broad Genomics, Northwest Genome Institute, Illumina, New York Genome Center, Baylor, and McDonnell Genome Institute) using Illumina X10 technology and DNA from blood. Here we report analyses from the 'Freeze 8' dataset where reads were aligned to human-genome build GRCh38 using a common pipeline across all sequencing centers. To perform variant quality control (QC) within the 'Freeze 8' dataset, a support vector machine (SVM) classifier was trained on known variant sites (positive labels) and Mendelian inconsistent variants (negative labels). Further variant filtering was done for variants with excess heterozygosity and Mendelian discordance. Sample QC measures included: concordance between annotated and inferred genetic sex, concordance between prior array genotype data and TOPMed WGS data, and pedigree checks. Details regarding the genotype 'freezes,' laboratory methods, data processing, and quality control are described on the TOPMed website and in a common document accompanying each study's dbGaP accession.

TOPMed structural variant calling and quality control

TOPMed structural variation (SV) callset release 1 was generated by Parliament2-muCNV pipeline across 138,134 multi-ethnic TOPMed WGS samples. The sample list overlaps largely with 'Freeze 8' callset except for the samples removed due to SV specific quality control issues. Parliament2 [6] is a multi-tool SV discovery pipeline that employs SV callers that have strengths in different SV types and sizes to maximize the detection sensitivity and accuracy. SVs detected by individual tools are then merged first across the callers and then across the samples using SURVIVOR [7] to generate a 'discovery' SV callset. The 'discovery' set is then genotyped and filtered by muCNV, a multi-sample SV genotyping software that performs joint genotyping based on multi-sample statistics across >100,000 samples [8]. Joint genotyping removes false discoveries by evaluating cluster separations using multi-sample distribution of read pair, split read, soft clips, and GC-corrected sequencing depth distributions. Parliament2, SURVIVOR, and muCNV are available for public access on GitHub:
https://github.com/slzarate/parliament2
https://github.com/fritzsedlazeck/SURVIVOR

https://github.com/gjun/muCNV

Analysis of Admixture

For RFMix inference, we combined samples with Native American ancestry in the Human Genome Diversity Project (HGDP) [9] and samples with African, East Asian, European and South Asian ancestries in the 1000 Genomes Project (1000G) [10]. We first retained variants that are available both in HGDP and in 1000G, then performed LD pruning using PLINK [11] with R^2 threshold of 0.01. ADMIXTURE [12] global ancestry analysis for HGDP samples identified 92 Native American samples with ≥90% Native American ancestry. To attain balanced sample size recommended for RFMix inference, we randomly selected 92 samples from each ancestry in the 1000G dataset.

Cohort Descriptions

BioMe

The Charles Bronfman Institute for Personalized Medicine at Mount Sinai Medical Center (MSMC), BioMe Biobank, founded in September 2007, is an ongoing, broadly-consented electronic health record-linked clinical care biobank that enrolls participants non-selectively from the Mount Sinai Medical Center patient population. The MSMC serves diverse local communities of upper Manhattan, including Central Harlem (86% African American), East Harlem (88% Hispanic/Latino), and Upper East Side (88% Caucasian/White) with broad health disparities.

JHS

The Jackson Heart Study (JHS, https://www.jacksonheartstudy.org/jhsinfo/) is a large, community-based, observational study whose participants were recruited from urban and rural areas of the three counties (Hinds, Madison and Rankin) that make up the Jackson, MS metropolitan statistical area (MSA). Participants were enrolled from each of 4 recruitment pools: random, 17%; volunteer, 30%; currently enrolled in the Atherosclerosis Risk in Communities (ARIC) Study, 31% and secondary family members, 22%. Recruitment was limited to non-institutionalized adult African Americans 35-84 years old, except in a nested family cohort where those 21 to 34 years of age were also eligible. The final cohort of 5,306 participants included 6.59% of all African American Jackson MSA residents aged 35-84 during the baseline exam (N-76,426, US Census 2000). Among these, approximately 3,700 gave consent that allows genetic research and deposition of data into dbGaP. Major components of three clinic examinations (Exam 1 – 2000-2004; Exam 2 – 2005-2008; Exam 3 – 2009-2013) include medical history, physical examination, blood/urine analytes and interview questions on areas such as: physical activity; stress, coping and spirituality; racism and discrimination; socioeconomic position; and access to health care. A fourth exam is ongoing. Extensive clinical phenotyping includes anthropometrics, electrocardiography, carotid ultrasound, ankle-brachial blood pressure index, echocardiography, CT chest and abdomen for coronary and aortic calcification, liver fat, and

subcutaneous and visceral fat measurement, and cardiac MRI. At 12-month intervals after the baseline clinic visit (Exam 1), participants have been contacted by telephone to: update information; confirm vital statistics; document interim medical events, hospitalizations, and functional status; and obtain additional sociocultural information. Questions about medical events, symptoms of cardiovascular disease and functional status are repeated annually. Ongoing cohort surveillance includes abstraction of medical records and death certificates for relevant International Classification of Diseases (ICD) codes and adjudication of nonfatal events and deaths. CMS data are currently being incorporated into the dataset.

MESA

The MESA study is a study of the characteristics of subclinical cardiovascular disease (disease detected non-invasively before it has produced clinical signs and symptoms) and the risk factors that predict progression to clinically overt cardiovascular disease or progression of subclinical disease. MESA researchers study a diverse, population-based sample of 6,814 asymptomatic men and women aged 45-84. Thirty-eight percent of the recruited participants are white, 28 percent African-American, 22 percent Hispanic, and 12 percent Asian, predominantly of Chinese descent. Participants were recruited from six field centers across the United States: Wake Forest University, Columbia University, Johns Hopkins University, University of Minnesota, Northwestern University and the University of California - Los Angeles.

WHI

The Women's Health Initiative (WHI) is a long-term, prospective, multi-center cohort study that investigates post-menopausal women's health 8. WHI was funded by the National Institutes of Health and the National Heart, Lung, and Blood Institute to study strategies to prevent heart disease, breast cancer, colon cancer, and osteoporotic fractures in women 50-79 years of age. WHI involves 161,808 women recruited between 1993 and 1998 at 40 centers across the US. The study consists of two parts: the WHI Clinical Trial which was a randomized clinical trial of hormone therapy, dietary modification, and calcium/Vitamin D supplementation, and the WHI Observational Study, which focused on many of the inequities in women's health research and provided practical information about the incidence, risk factors, and interventions related to heart disease, cancer, and osteoporotic fractures.

Acknowledgements

TOPMed Accession #	TOPMed Project	Parent Study Name	TOPMed Phase	Omics Center	Omics Support
phs001644	BioMe	BioMe	3	Baylor	HHSN268201600033I
phs001644	BioMe	BioMe	3	MGI	HHSN268201600037I
phs000964	JHS	JHS	1	NWGC	HHSN268201100037C

phs001416	AA_CAC	MESA AA_CAC	2	Broad Genomics	HHSN268201500014C
phs001416	MESA	MESA	2	Broad Genomics	3U54HG003067-13S1
phs001237	WHI	WHI	2	Broad Genomics	HHSN268201500014C

BioMe: The Mount Sinai BioMe Biobank has been supported by The Andrea and Charles Bronfman Philanthropies and in part by Federal funds from the NHLBI and NHGRI (U01HG00638001; U01HG007417; X01HL134588). We thank all participants in the Mount Sinai Biobank. We also thank all our recruiters who have assisted and continue to assist in data collection and management and are grateful for the computational resources and staff expertise provided by Scientific Computing at the Icahn School of Medicine at Mount Sinai.

JHS: The Jackson Heart Study (JHS) is supported and conducted in collaboration with Jackson State University (HHSN268201800013I), Tougaloo College (HHSN268201800014I), the Mississippi State Department of Health (HHSN268201800015I) and the University of Mississippi Medical Center (HHSN268201800010I, HHSN268201800011I and HHSN268201800012I) contracts from the National Heart, Lung, and Blood Institute (NHLBI) and the National Institute on Minority Health and Health Disparities (NIMHD). The authors also wish to thank the staffs and participants of the JHS.

The views expressed in this manuscript are those of the authors and do not necessarily represent the views of the National Heart, Lung, and Blood Institute; the National Institutes of Health; or the U.S. Department of Health and Human Services.

MESA: MESA and the MESA SHARe project are conducted and supported by the National Heart, Lung, and Blood Institute (NHLBI) in collaboration with MESA investigators. Support for MESA is provided by contracts 75N92020D00001, HHSN268201500003I, N01-HC-95159, 75N92020D00005, N01-HC-95160, 75N92020D00002, N01-HC-95161, 75N92020D00003, N01-HC-95162, 75N92020D00006, N01-HC-95163, 75N92020D00004, N01-HC-95164, 75N92020D00007, N01-HC-95165, N01-HC-95166, N01-HC-95167, N01-HC-95168, N01-HC-95169, UL1-TR-000040, UL1-TR-001079, UL1-TR-001420. MESA Family is conducted and supported by the National Heart, Lung, and Blood Institute (NHLBI) in collaboration with MESA investigators. Support is provided by grants and contracts R01HL071051, R01HL071205, R01HL071250, R01HL071251, R01HL071258, R01HL071259, by the National Center for Research Resources, Grant UL1RR033176. The provision of genotyping data was supported in part by the National Center for Advancing Translational Sciences, CTSI grant UL1TR001881, and the National Institute of Diabetes and Digestive and Kidney Disease Diabetes Research Center (DRC) grant DK063491 to the Southern California Diabetes Endocrinology Research Center.

WHI: The WHI program is funded by the National Heart, Lung, and Blood Institute, National Institutes of Health, U.S. Department of Health and Human Services through contracts

HHSN268201600018C, HHSN268201600001C, HHSN268201600002C, HHSN268201600003C, and HHSN268201600004C.

The views expressed in this manuscript are those of the authors and do not necessarily represent the views of the National Heart, Lung, and Blood Institute; the National Institutes of Health; or the U.S. Department of Health and Human Services.

References

1. Gottesman, O., et al., *The Electronic Medical Records and Genomics (eMERGE) Network: past, present, and future.* Genet Med, 2013. **15**(10): p. 761-71.
2. Taylor, H.A., Jr., et al., *Toward resolution of cardiovascular health disparities in African Americans: design and methods of the Jackson Heart Study.* Ethn Dis, 2005. **15**(4 Suppl 6): p. S6-4-17.
3. Wilson, J.G., et al., *Study design for genetic analysis in the Jackson Heart Study.* Ethn Dis, 2005. **15**(4 Suppl 6): p. S6-30-37.
4. Bild, D.E., et al., *Multi-Ethnic Study of Atherosclerosis: objectives and design.* Am J Epidemiol, 2002. **156**(9): p. 871-81.
5. *Design of the Women's Health Initiative clinical trial and observational study. The Women's Health Initiative Study Group.* Control Clin Trials, 1998. **19**(1): p. 61-109.
6. Zarate, S., et al., *Parliament2: Accurate structural variant calling at scale.* Gigascience, 2020. **9**(12).
7. Jeffares, D.C., et al., *Transient structural variations have strong effects on quantitative traits and reproductive isolation in fission yeast.* Nat Commun, 2017. **8**: p. 14061.
8. Jun, G., et al., *muCNV: Genotyping Structural Variants for Population-level Sequencing.* Bioinformatics, 2021.
9. Li, J.Z., et al., *Worldwide human relationships inferred from genome-wide patterns of variation.* Science, 2008. **319**(5866): p. 1100-4.
10. Genomes Project, C., et al., *A global reference for human genetic variation.* Nature, 2015. **526**(7571): p. 68-74.
11. Chang, C.C., et al., *Second-generation PLINK: rising to the challenge of larger and richer datasets.* Gigascience, 2015. **4**: p. 7.
12. Alexander, D.H., J. Novembre, and K. Lange, *Fast model-based estimation of ancestry in unrelated individuals.* Genome Res, 2009. **19**(9): p. 1655-64.
