# Supplementary material for: TOP-LD: A tool to explore linkage disequilibrium with TOPMed whole-genome sequence data
Source: Am J Hum Genet. 2022 May 2;109(6):1175–81. doi: 10.1016/j.ajhg.2022.04.006 (PMC9247832; doi:10.1016/j.ajhg.2022.04.006)
Supplement: Document S2. Article plus supplemental information [file mmc2.pdf]

TOP-LD: A tool to explore linkage disequilibrium with TOPMed whole-genome sequence data

Authors

Le Huang, Jonathan D. Rosen, Quan Sun, ...,
Alex P. Reiner, Paul L. Auer, Yun Li

Correspondence

pauer@mcw.edu (P.L.A.),
yunli@med.unc.edu (Y.L.)

Huang et al., 2022, The American Journal of Human Genetics *109*, 1175–1181
June 2, 2022 © 2022 The Authors.
https://doi.org/10.1016/j.ajhg.2022.04.006

CellPress

TOP-LD: A tool to explore linkage disequilibrium with TOPMed whole-genome sequence data

Le Huang,[1,19] Jonathan D. Rosen,[2,19] Quan Sun,[2,19] Jiawen Chen,[2] Marsha M. Wheeler,[3] Ying Zhou,[4] Yuan-I Min,[5] Charles Kooperberg,[4] Matthew P. Conomos,[6] Adrienne M. Stilp,[6] Stephen S. Rich,[7] Jerome I. Rotter,[8] Ani Manichaikul,[7] Ruth J.F. Loos,[9,10] Eimear E. Kenny,[9] Thomas W. Blackwell,[11] Albert V. Smith,[11] Goo Jun,[12] Fritz J. Sedlazeck,[13] Ginger Metcalf,[13] Eric Boerwinkle,[14] NHLBI Trans-Omics for Precision Medicine (TOPMed) Consortium, Laura M. Raffield,[15] Alex P. Reiner,[16,4] Paul L. Auer,[17,*] and Yun Li[2,15,18,*]

Summary

Current publicly available tools that allow rapid exploration of linkage disequilibrium (LD) between markers (e.g., HaploReg and LDlink) are based on whole-genome sequence (WGS) data from 2,504 individuals in the 1000 Genomes Project. Here, we present TOP-LD, an online tool to explore LD inferred with high-coverage (~30×) WGS data from 15,578 individuals in the NHLBI Trans-Omics for Precision Medicine (TOPMed) program. TOP-LD provides a significant upgrade compared to current LD tools, as the TOPMed WGS data provide a more comprehensive representation of genetic variation than the 1000 Genomes data, particularly for rare variants and in the specific populations that we analyzed. For example, TOP-LD encompasses LD information for 150.3, 62.2, and 36.7 million variants for European, African, and East Asian ancestral samples, respectively, offering 2.6- to 9.1-fold increase in variant coverage compared to HaploReg 4.0 or LDlink. In addition, TOP-LD includes tens of thousands of structural variants (SVs). We demonstrate the value of TOP-LD in fine-mapping at the *GGT1* locus associated with gamma glutamyltransferase in the African ancestry participants in UK Biobank. Beyond fine-mapping, TOP-LD can facilitate a wide range of applications that are based on summary statistics and estimates of LD. TOP-LD is freely available online.

Linkage disequilibrium (LD), i.e., the non-random association of alleles at different variant sites in a given population, is an important genetic phenomenon. Patterns of LD between genetic markers can be leveraged to gain insights in a variety of different applications, from population genetic research to disease association studies.[1,2] With the growth of whole-genome sequencing (WGS) and high-throughput array and genotype imputation technologies, resources for calculating LD across populations have expanded to encompass multiple populations at variant sites with increasingly rare frequencies.[3-6] Due to the centrality of LD in a host of applications, multiple tools exist for querying LD between genetic markers in different populations. The current most widely used LD lookup tools, HaploReg[7] and LDlink,[8] base their LD estimates on the 1000 Genomes data. Specifically, HaploReg uses phase 1 and LDlink uses phase 3 1000 Genomes data. Although the 1000 Genomes data contains LD information on >99% of genetic markers with minor allele frequency (MAF) > 1% in a variety of populations,[4] there remains a dearth of publicly available information on LD between markers with MAF < 1%. We have created a new LD lookup tool (called "TOP-LD"), in the spirit of HaploReg and LDlink, that is based on deep (30×) WGS data from the NHLBI Trans-Omics for Precision Medicine (TOPMed) Program. Because the TOPMed data contain much larger sample sizes with greater depth of sequencing than the 1000 Genomes project, TOP-LD provides a significant upgrade in LD information availability, specifically by including single-nucleotide variants and small indels (referred to hereafter simply as "SNVs") with MAF < 1% as well as structural variants (SVs). Here, we describe the data and

[1]Curriculum in Bioinformatics and Computational Biology, University of North Carolina at Chapel Hill, Chapel Hill, NC 27599, USA; [2]Department of Biostatistics, University of North Carolina at Chapel Hill, Chapel Hill, NC 27599, USA; [3]Department of Genome Sciences, University of Washington, Seattle, WA 98105, USA; [4]Division of Public Health Sciences, Fred Hutchinson Cancer Research Center, Seattle, WA 98109, USA; [5]Department of Medicine, University of Mississippi Medical Center, Jackson, MS 39216, USA; [6]Department of Biostatistics, University of Washington, Seattle, WA 98105, USA; [7]Center for Public Health Genomics, Department of Public Health Sciences, University of Virginia School of Medicine, Charlottesville, VA 22908, USA; [8]The Institute for Translational Genomics and Population Sciences, Department of Pediatrics, The Lundquist Institute for Biomedical Innovation at Harbor-UCLA Medical Center, Torrance, CA 90502, USA; [9]The Charles Bronfman Institute for Personalized Medicine, Icahn School of Medicine at Mount Sinai, New York City, NY 10029, USA; [10]Novo Nordisk Foundation Center for Basic Metabolic Research, Faculty of Health and Medical Sciences, University of Copenhagen, 2200 Copenhagen, Denmark; [11]TOPMed Informatics Research Center, University of Michigan, Department of Biostatistics, Ann Arbor, MI 48109, USA; [12]Human Genetics Center, School of Public Health, The University of Texas Health Science Center at Houston, Houston, TX 77030, USA; [13]Human Genome Sequencing Center, Baylor College of Medicine, Houston, TX 77030, USA; [14]Human Genetics Center, Department of Epidemiology, Human Genetics, and Environmental Sciences, School of Public Health, The University of Texas Health Science Center at Houston, Houston, TX 77030, USA; [15]Department of Genetics, University of North Carolina at Chapel Hill, Chapel Hill, NC 27599, USA; [16]Department of Epidemiology, University of Washington, Seattle, WA 98195, USA; [17]Division of Biostatistics, Institute for Health and Equity, and Cancer Center, Medical College of Wisconsin, Milwaukee, WI 53226, USA; [18]Department of Computer Science, University of North Carolina at Chapel Hill, Chapel Hill, NC 27599, USA
[19]These authors contributed equally
*Correspondence: pauer@mcw.edu (P.L.A.), yunli@med.unc.edu (Y.L.)
https://doi.org/10.1016/j.ajhg.2022.04.006.
© 2022 The Authors. This is an open access article under the CC BY license (http://creativecommons.org/licenses/by/4.0/).

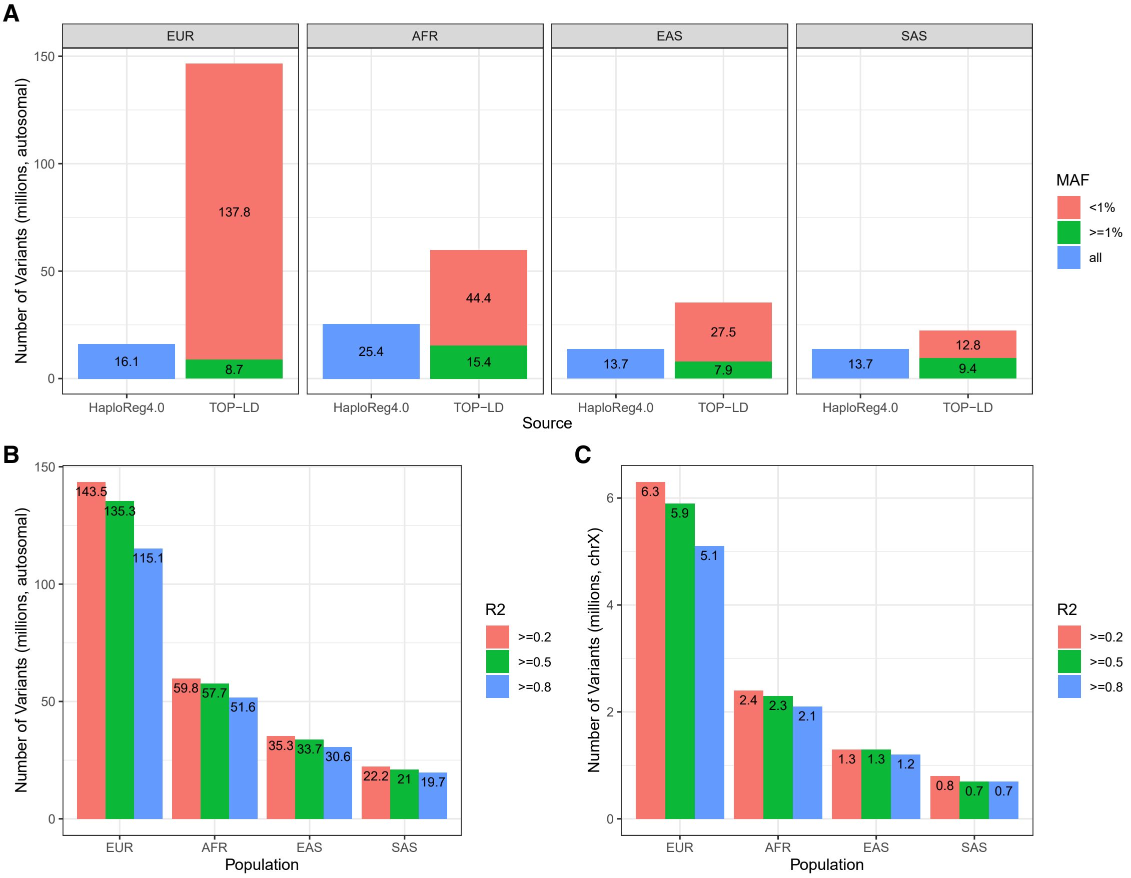

Figure 1. Number of variants included in TOP-LD
(A) Comparison of autosomal variants with HaploReg 4.0 by population. Blue bars on the left show total number of autosomal variants in HaploReg4.0. Green and red indicate common (MAF $\geq$ 1%) and uncommon (MAF $<$ 1%) autosomal variants in TOP-LD. Note that HaploReg4.0 provides LD for ASN (Asian) with no separate information for EAS and SAS. Therefore, we used the same 13.7 million ASN variants for comparison in both EAS and SAS.
(B) Number of autosomal variants in TOP-LD breaking down by LD R^2 threshold. The majority of the variants have at least one LD proxy with $R^2 \geq 0.8$.
(C) Number of chrX variants in TOP-LD breaking down by LD R^2 threshold.
(Note: LD information downloaded from HaploReg4.0 does not contain chromosome X. Therefore, we compared TOP-LD with HaploReg4.0 only for autosomal variants).

methods that went into creating TOP-LD along with specific examples of how TOP-LD can provide essential information that is missed by HaploReg and LDlink.

We used TOPMed WGS data[6] from the following four cohorts: BioMe Biobank (BioMe), the Multi-Ethnic Study of Atherosclerosis (MESA), the Jackson Heart Study (JHS), and the Women's Health Initiative (WHI). We aimed to provide LD estimates for genetically homogeneous groups of individuals from one of the following four ancestral populations: European (EUR), African (AFR), East Asian (EAS), and South Asian (SAS). To select appropriate samples, we first inferred local and global ancestry for all participants in these four cohorts by using RFMix,[9] with reference populations including five ancestral groups, namely African, Native American, East Asian, European, and South Asian. After local ancestry inference, we then retained only TOPMed samples with >90% estimated ancestry from a single population, as estimated via RFMix. We further removed related individuals by using a stringent kinship coefficient threshold of $2^{-5.5}$ obtained via PC-Relate.[10] This threshold of $2^{-5.5}$ removes pairs within as far as fifth degree relationship. The final dataset included 1,335 unrelated individuals of African, 844 of East Asian, 13,160 of European, and 239 of South Asian ancestry for pairwise LD inference. Regarding variants, we started with all TOPMed freeze 8 polymorphic variants that passed quality control and retained multi-allelic variants or multiple entries at the same position, resulting in a total of 23.0–153.0 million SNVs in each of the ancestral groups (Figure 1A, Table S1).

We inferred LD separately within each of the four ancestral groups, for all pairs of variants within 1 Mb of each other, and retained LD pairs meeting a minimum R^2 threshold of 0.2. The reported R^2 between two variants is the squared Pearson correlation coefficient between their phased haplotypes, where phasing was performed with Eagle 2.4 for all polymorphic variants, similar to phasing of the freeze 5 data.[6] No minimum minor allele count thresholding was used, that

Table 1. Summary of SVs by population

Population	Number of SVs	Number of SVs in LD w/SNVs[a]	Number of SVs with MAF < 0.01
EUR	79,004	16,301	69,011
AFR	44,859	15,151	27,978
SAS	16,511	10,392	7,292
EAS	20,789	7,498	12,902

[a]Number of SVs having at least one SNV LD tag with $R^2 \geq 0.8$.

is, even singletons in our sample were included in LD calculations. We also report the direction of each association as either positive (+) or negative (−) on the basis of the sign of the Pearson correlation coefficient between the corresponding pair of reference (REF) alleles. In addition to R^2, we also report D-prime statistics for each pair of variants meeting the R^2 of 0.2.

We filtered chromosome X to exclude the pseudo-autosomal regions: PAR1 (bp 10,001–2,781,479, GRCh38) and PAR2 (bp 155,701,383–156,030,895, GRCh38). Variants that were not coded as homozygous in the males were excluded from the LD calculations. We inferred LD for the remaining variants by using a total of 2F + M haplotypes, where F and M are the numbers of females and males, respectively.

The TOPMed structural variant (SV) call-set freeze 1 was merged with a reduced TOPMed SNV call-set where SNVs with MAF < 0.1% were filtered out before merging, and then the merged SV-SNV dataset was phased with Eagle2.[11] SVs with >10% missingness were removed prior to phasing. For each ancestry group, we included 16.5–79K SVs (deletions, duplications, and inversion) with the majority being lower frequency (e.g., 7–69K with MAF < 1%) (Table 1). LD values were subsequently estimated as the squared Pearson correlation coefficient between the corresponding pair of phased alleles.

TOPMed LD information was then loaded into the TOP-LD website, which is powered by a combination of MySQL, PHP, Javascript, and Apache2 under the CloudSQL and Compute Engine of Google Cloud Platform. The web interface provides access to all precomputed LD estimates. Users have the option to either paste or upload a file containing variant(s) of interest. Users can specify the population (East Asian, European, African, or South Asian) in which LD was estimated. In TOP-LD, markers are identified by rsID, or chr:position, or chr:position:REF:ALT for SNVs, or TOPMed variant names for SVs (in the format of DEL/DUP/INV_chr:startPosition-endPosition, for example, DEL_10:85001–97300). TOP-LD returns all variants within a pre-specified LD threshold (ranging from R^2 values of 0.2 to 1.0) with the query variant. TOP-LD supports fast batch queries (Figure 2); querying a single variant takes ~0.5 s, while a batch query of 500 variants takes ~2.3 seconds. TOP-LD currently allows a maximum of 500 variants in one query.

After submitting the query, the website auto-directs to a result page that contains two parts: LD information on the top panel and variant information on the bottom panel. The latter provides basic information for the queried variants, including position, marker name, alleles (REF and ALT), and minor allele frequency (MAF). Markers not in the database will have "none" for all fields except marker names. The LD panel displays related LD metrics, one pair of variants on each line, including both R^2, D′, and the sign of LD (measured between REF alleles of the two variants), along with marker name, marker position,

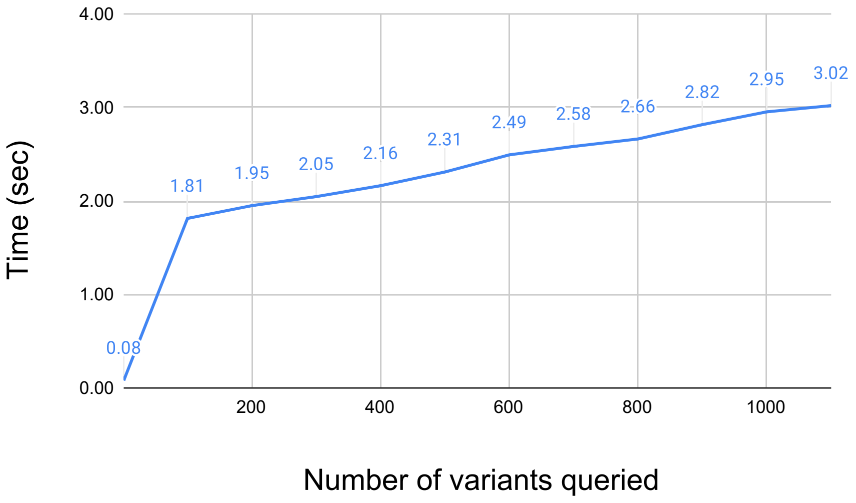

Figure 2. Elapsed time (in seconds) for queries
The x axis represents the number of variants queried, and the y axis represents the elapsed time.

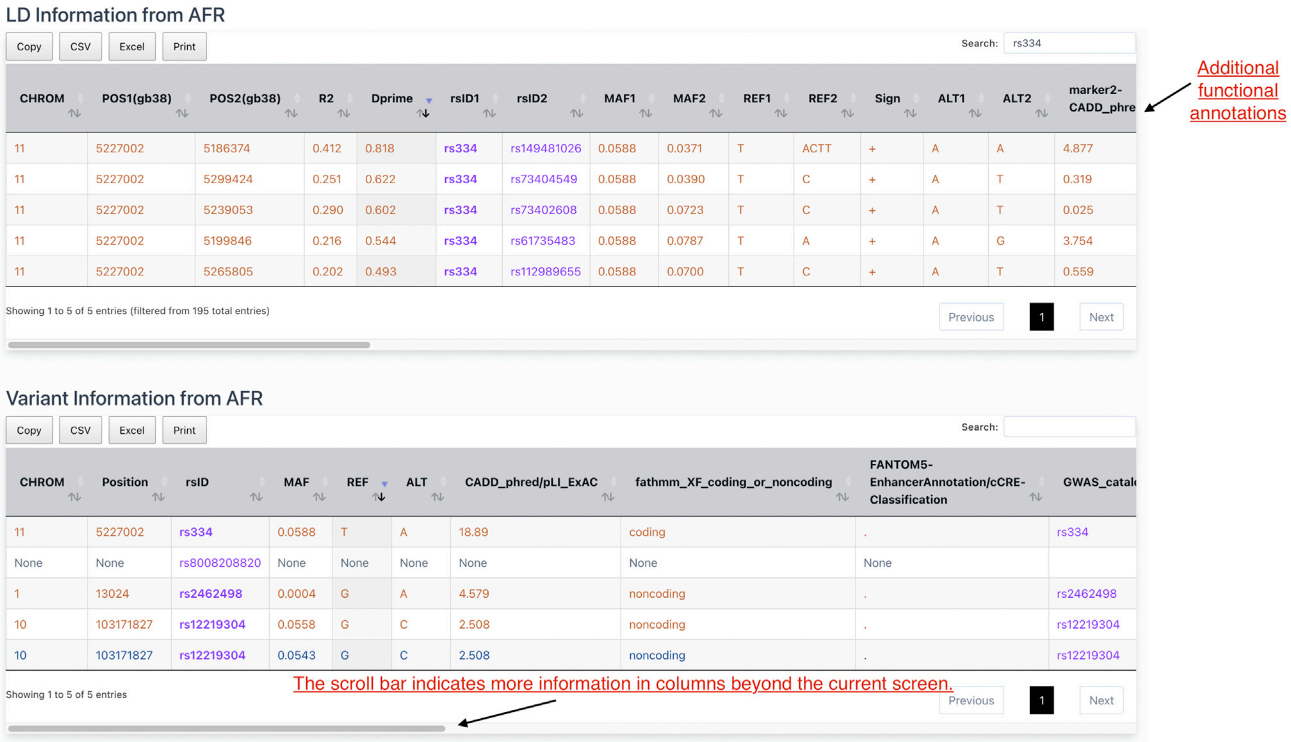

LD Information from AFR

Copy | CSV | Excel | Print   Search: rs334

CHROM	POS1(gb38)	POS2(gb38)	R2	Dprime	rsID1	rsID2	MAF1	MAF2	REF1	REF2	Sign	ALT1	ALT2	marker2-CADD_phre
11	5227002	5186374	0.412	0.818	rs334	rs149481026	0.0588	0.0371	T	ACTT	+	A	A	4.877
11	5227002	5299424	0.251	0.622	rs334	rs73404549	0.0588	0.0390	T	C	+	A	T	0.319
11	5227002	5239053	0.290	0.602	rs334	rs73402608	0.0588	0.0723	T	C	+	A	T	0.025
11	5227002	5199846	0.216	0.544	rs334	rs61735483	0.0588	0.0787	T	A	+	A	G	3.754
11	5227002	5265805	0.202	0.493	rs334	rs112989655	0.0588	0.0700	T	C	+	A	T	0.559

Showing 1 to 5 of 5 entries (filtered from 195 total entries)   Previous 1 Next

Variant Information from AFR

Copy | CSV | Excel | Print   Search:

CHROM	Position	rsID	MAF	REF	ALT	CADD_phred/pLI_ExAC	fathmm_XF_coding_or_noncoding	FANTOM5-EnhancerAnnotation/cCRE-Classification	GWAS_catal
11	5227002	rs334	0.0588	T	A	18.89	coding	.	rs334
None	None	rs8008208820	None	None	None	None	None	None	
1	13024	rs2462498	0.0004	G	A	4.579	noncoding	.	rs2462498
10	103171827	rs12219304	0.0558	G	C	2.508	noncoding	.	rs12219304
10	103171827	rs12219304	0.0543	G	C	2.508	noncoding	.	rs12219304

Showing 1 to 5 of 5 entries   Previous 1 Next

Figure 3. An example query result
The result contains two parts. The top part "LD information from AFR" shows the LD information where each line provides information between a query variant (rsID1) and one of its corresponding LD proxies (rsID2). The bottom part "variant information from AFR" provides variant information, which shows basic information for each query variant. From the bottom part, we know that the user's query includes four variants: rs334, rs8008208820, rs2462498, and rs12219304. Variants not included in LD calculation will have "none" records. For instance, rs8008208820 in this example query is not involved in LD inference and therefore will not have any LD proxies in the top part simply because of no data. Records from SV inference are in blue and those from SNV data are in orange. Some variants may appear twice because they are included in both SNV LD calculation and SV calculation. For example, in this example, rs12219304 appeared twice with MAF 0.0558 from the SNV source (second last record in orange) and MAF 0.0543 from the SV source (last record in blue).

alleles, and frequency for both variants in the pair (Figure 3). In addition, we provide the following pieces of information for SNVs from WGSA annotation[12]: CADD score (phred-scaled), fathmm_XF_coding_or_noncoding classification, FANTOM5 enhancer annotations, gene name, and relative location to gene as well as a link to GWAS catalog query results.[13] For SVs, we provide a variety of annotations including gene(s) overlapping the SV, the SV's location relative to gene, the gene's pLI score, overlapping candidate *cis*-regulatory regions (cCREs) from ENCODE SCREEN.[14,15] The query results can be sorted, searched, copied, exported, and printed for further analyses.

The TOP-LD tool leverages TOPMed WGS data, whose much larger sample size and high depth sequencing lead to LD information for a much larger number of variants compared to the 1000 Genomes Project. As shown in Figure 1A and Table S1, TOP-LD offers 2.6- to 9.1-fold increase in variant coverage compared to the other state-of-the-art resources such as HaploReg 4.0 or LDlink. For example, for the European population, TOP-LD includes 146.5 million autosomal SNVs, while HaploReg 4.0 or LDlink contains 16.1 million variants. Not surprisingly, the vast majority of the variants in TOP-LD that are not in 1000 Genomes, contributing to the up to 9.1× increase, are low frequency or rare. For example, out of the 146.5 million autosomal SNVs cataloged in the TOP-LD European population, 137.8 million have MAF < 0.01 (Figure 1A, Table S1). Most of the variants have LD proxies. For example, 115.1 out of the 146.5 (78.6%) million autosomal variants have at least one LD tag with $R^2 \geq 0.8$ and if we further relax the R^2 threshold to 0.5 and 0.2, the number increases to 135.3 (92.4%) and 143.5 (98.0%), respectively (Figure 1B).

For chromosome X, we have included 6.5 million, 2.4 million, 1.3 million, and 760,000 variants for the European, African, East Asian, and South Asian populations, respectively (Table S1). Similar to the autosomal variants, the majority of these variants have at least one LD proxy with $R^2 \geq 0.8$: 5.1 million, European; 2.1 million, African; 1.2 million East Asian; 690,000, South Asian (Figure 1C, Table S2).

To evaluate the consistency between TOP-LD estimates and those from Haploreg v4.1, we collected the set of overlapping variants based on rsID with MAF ≥ 0.05 for Europeans and Africans. This set of variants was further filtered

Table 2. Summary statistics of distinct working truth at *GGT1* locus associated with gamma glutamyltransferase

Signal	Variant	Position (hg38)	Effect allele	Unconditional p value	p value conditional on previous signals[a]	Effect allele frequency
1	rs4049904	24609759	G	2.82e−61	N/A	10.27%
2	rs73404962	24598530	G	4.46e−29	2.00e−36	5.63%
3	rs743369	24588099	A	9.94e−36	7.51e−27	11.94%
4	rs6004193	24598329	C	4.23e−41	3.25e−19	18.27%
5	rs57719575	24609020	C	3.97e−38	1.98e−24	14.86%
6	rs3876101	24607291	A	2.66e−15	1.17e−13	35.45%
7	rs116161010	24585912	T	5.69e−17	7.70e−9	7.13%

[a]The p values are reported from the sequential conditional analysis. For example, we report the p value for rs73404962 conditional on rs4049904, the p value of rs743369 conditional on both rs4049904 and rs73404962, and so forth.

such that the MAF values were within 10% of each other because large MAF differences would induce large LD differences. Figures S1 and S2 show high level of agreement between TOP-LD and Haploreg v4.1 LD estimates (e.g., Pearson correlation = 0.972 and 0.962 for European and African chromosome 1, respectively). Similarly, comparison of the chromosome X TOP-LD estimates for females and males again show high level of consistency (Pearson correlation = 0.992 and 0.975 for European and African population, respectively) (Figures S3 and S4).

To demonstrate the utility of TOP-LD, we performed fine-mapping at the *GGT1* locus on chromosome 22, which is known to be associated with gamma glutamyltransferase.[16] We performed sequential conditional analysis with EPACTS[17] by using individual-level data among 8,768 UK Biobank participants of African ancestry following the same strategy in our previous work[18] adjusting for the same covariates as in Sun et al.[19] The sequential conditional analyses with individual-level data identified seven distinct signals at the *GGT1* locus associated with gamma glutamyltransferase (Table 2). Because we used individual-level data for this conditional analysis, we considered these seven distinct signals to be the "working truth."

We then carried out fine-mapping analysis with the FINEMAP method[20] by using only GWAS summary statistics from Sun et al.[19] We applied FINEMAP with an LD reference either from TOP-LD or from the 1000 Genomes Project and assessed the performance by comparing the results with "working truth" established from the sequential conditional analysis of the individual-level data.

FINEMAP produced 95% credible sets containing five variants when using either the 1000 Genomes (1000G) Project LD panel or the TOP-LD panel (see Table 3). However, the 1000G-based credible set contained only one of the seven signals from the "working truth" set. In contrast, the TOP-LD-based credible set contained three of the seven signals from the "working truth" set. In addition, because the lead variant from each conditional analysis (corresponding to each distinct signal) is selected somewhat arbitrarily, we also considered their LD proxies. When we considered any LD proxy (using a lenient R^2 threshold of 0.2) of a variant in the working truth set, the 1000G-based results still only identified a single signal from the working truth, whereas the TOP-LD-based results identified four of the seven signals (Table 3).

We also used TOP-LD to aid in the identification and prioritization of potentially causal structural variants at GWAS loci. For example, our recent association analysis[21] with TOPMed data identified an African-specific (MAF = 0.129) variant rs28450540 associated with lower monocyte count ($p = 3.65 \times 10^{-17}$). Query for LD tags via TOP-LD revealed a ~600 bp deletion near *S1PR3* in perfect LD ($R^2 = 1$) with rs28450540 in the African population. We performed genome editing in monocytic and primary human HSPCs followed by xenotransplantation, which provides evidence that the deletion disrupts an *S1PR3* monocyte enhancer leading to decreased *S1PR3* expression. These preliminary data from functional experiments suggest that the 600 bp deletion is most likely casual but would have been missed in standard association analysis with

Table 3. FINEMAP credible-set variants

		Variant 1	Variant 2	Variant 3	Variant 4	Variant 5
1000G reference	credible-set variant	rs4049904	rs147866692	rs570263050	rs115231893	22:24649848:G:A (hg38)
	LD with working truth	1 (w/rs4049904 itself)	0.464 (w/ rs4049904)	0.606 (w/rs4049904)	0.275 (w/ rs4049904)	0.434 (w/rs4049904)
TOP-LD reference	credible-set variant	rs4049904	rs743369	rs57719575	rs2073397	rs5751902
	LD with working truth	1 (w/rs4049904 itself)	1 (w/rs743369 itself)	1 (w/rs57719575 itself)	0.83 (w/rs6004193)	0.51 (w/rs6004193)

The two five-variant credible sets provided by FINEMAP with either 1000G or TOP-LD as reference. For each credible-set variant, we list the corresponding variant (and the LD Rsq) from the working truth that has the highest LD.

only SNVs.[22] TOP-LD offers a simple and efficient approach to rescue such putative causal structural variants.

LD information, reflecting recombination, natural selection, and demographic history, has always been of intense interest in population genetics and complex trait association studies. LD information is also indispensable for a wide range of other applications, including GWAS follow-up and many summary-statistics-based inferences including fine-mapping, imputation of association summary statistics, construction of polygenic risk scores (PRSs), and interpretation and prioritization of GWAS results for further functional and clinical studies. TOP-LD significantly boosts the coverage of lower frequency variants by harnessing the power of high-coverage (~30×) WGS data of over 15,000 individuals primarily of a single continental ancestry. We demonstrate the utility of TOP-LD in fine-mapping at the *GGT1* locus and variant prioritization at the *S1PR3* locus. The LD information provided by TOP-LD will facilitate a range of essential inferences for common and rare variation across a diverse range of populations.

Data and code availability

Data generated for this study can be accessed via the TOP-LD web portal: http://topld.genetics.unc.edu.

Supplemental information

Supplemental information can be found online at https://doi.org/10.1016/j.ajhg.2022.04.006.

Acknowledgments

We gratefully acknowledge the studies and participants who provided biological samples and data for TOPMed. The TOPMed Banner Authorship list can be found at: https://www.nhlbiwgs.org/topmed-banner-authorship. The project described is supported by funding from the National Institutes of Health through R01HL129132 and U01HG011720 (Y.L.), R01HL146500 (A.P.R.), and KL2TR002490 (L.M.R.). Y.L., J.D.R., and L.H. are also partially supported by R01HL146500, U24 AR076730, and U01DA052713. The content is solely the responsibility of the authors and does not necessarily represent the official views of the NIH.

Declaration of interests

L.M.R. is a consultant for the TOPMed Administrative Coordinating Center (through Westat).

Received: December 20, 2021
Accepted: April 8, 2022
Published: May 2, 2022

Web resources

EPACTS, https://genome.sph.umich.edu/wiki/EPACTS

FINEMAP, http://www.christianbenner.com/

HaploReg, https://pubs.broadinstitute.org/mammals/haploreg/

LDlink, https://ldlink.nci.nih.gov/

TOP-LD, http://topld.genetics.unc.edu/

TOPMed, https://topmed.nhlbi.nih.gov/

References

1. Slatkin, M. (2008). Linkage disequilibrium—understanding the evolutionary past and mapping the medical future. Nat. Rev. Genet. *9*, 477–485. https://doi.org/10.1038/nrg2361.
2. Bush, W.S., and Moore, J.H. (2012). Chapter 11: Genome-wide association studies. PLoS Comput. Biol. *8*, e1002822. https://doi.org/10.1371/journal.pcbi.1002822.
3. Choudhury, A., Aron, S., Botigué, L.R., Sengupta, D., Botha, G., Bensellak, T., Wells, G., Kumuthini, J., Shriner, D., Fakim, Y.J., et al. (2020). High-depth African genomes inform human migration and health. Nature *586*, 741–748. https://doi.org/10.1038/s41586-020-2859-7.
4. The 1000 Genomes Project Consortium (2015). A global reference for human genetic variation. Nature *526*, 68–74.
5. Consortium, I.H., Gibbs, R.A., Peltonen, L., Altshuler, D.M., Gibbs, R.A., Peltonen, L., Dermitzakis, E., Schaffner, S.F., Yu, F., Peltonen, L., et al. (2010). Integrating common and rare genetic variation in diverse human populations. Nature *467*, 52–58. https://doi.org/10.1038/nature09298.
6. Taliun, D., Harris, D.N., Kessler, M.D., Carlson, J., Szpiech, Z.A., Torres, R., Taliun, S.A.G., Corvelo, A., Gogarten, S.M., Kang, H.M., et al. (2021). Sequencing of 53,831 diverse genomes from the NHLBI TOPMed Program. Nature *590*, 290–299. https://doi.org/10.1038/s41586-021-03205-y.
7. Ward, L.D., and Kellis, M. (2016). HaploReg v4: systematic mining of putative causal variants, cell types, regulators and target genes for human complex traits and disease. Nucleic Acids Res. *44*, D877–D881. https://doi.org/10.1093/nar/gkv1340.
8. Machiela, M.J., and Chanock, S.J. (2015). LDlink: a web-based application for exploring population-specific haplotype structure and linking correlated alleles of possible functional variants. Bioinformatics (Oxford, England) *31*, 3555–3557. https://doi.org/10.1093/bioinformatics/btv402.
9. Maples, B.K., Gravel, S., Kenny, E.E., and Bustamante, C.D. (2013). RFMix: a discriminative modeling approach for rapid and robust local-ancestry inference. Am. J. Hum. Genet. *93*, 278–288. https://doi.org/10.1016/j.ajhg.2013.06.020.
10. Conomos, M.P., Reiner, A.P., Weir, B.S., and Thornton, T.A. (2016). Model-free estimation of recent genetic relatedness. Am. J. Hum. Genet. *98*, 127–148. https://doi.org/10.1016/j.ajhg.2015.11.022.
11. Loh, P.R., Danecek, P., Palamara, P.F., Fuchsberger, C., A Reshef, Y., K Finucane, H., Schoenherr, S., Forer, L., McCarthy, S., Abecasis, G.R., et al. (2016). Reference-based phasing using the haplotype reference consortium panel. Nat. Genet. *48*, 1443–1448. https://doi.org/10.1038/ng.3679.
12. Liu, X., White, S., Peng, B., Johnson, A.D., Brody, J.A., Li, A.H., Huang, Z., Carroll, A., Wei, P., Gibbs, R., et al. (2016). WGSA: an annotation pipeline for human genome sequencing studies. J. Med. Genet. *53*, 111–112. https://doi.org/10.1136/jmedgenet-2015-103423.
13. MacArthur, J., Bowler, E., Cerezo, M., Gil, L., Hall, P., Hastings, E., Junkins, H., McMahon, A., Milano, A., Morales, J., et al.

(2017). The new NHGRI-EBI Catalog of published genome-wide association studies (GWAS Catalog). Nucleic Acids Res. *45*. D896–d901. https://doi.org/10.1093/nar/gkw1133.
14. Geoffroy, V., Herenger, Y., Kress, A., Stoetzel, C., Piton, A., Dollfus, H., and Muller, J. (2018). AnnotSV: an integrated tool for structural variations annotation. Bioinformatics (Oxford, England) *34*, 3572–3574. https://doi.org/10.1093/bioinformatics/bty304.
15. Moore, J.E., Moore, J.E., Purcaro, M.J., Pratt, H.E., Epstein, C.B., Shoresh, N., Adrian, J., Kawli, T., Davis, C.A., Dobin, A., et al. (2020). Expanded encyclopaedias of DNA elements in the human and mouse genomes. Nature *583*, 699–710. https://doi.org/10.1038/s41586-020-2493-4.
16. Pazoki, R., Vujkovic, M., Elliott, J., Evangelou, E., Gill, D., Ghanbari, M., van der Most, P.J., Pinto, R.C., Wielscher, M., Farlik, M., et al. (2021). Genetic analysis in European ancestry individuals identifies 517 loci associated with liver enzymes. Nat. Commun. *12*, 2579. https://doi.org/10.1038/s41467-021-22338-2.
17. Kang, H.M., Sul, J.H., Service, S.K., Zaitlen, N.A., Kong, S.Y., Freimer, N.B., Sabatti, C., and Eskin, E. (2010). Variance component model to account for sample structure in genome-wide association studies. Nat. Genet. *42*, 348–354. https://doi.org/10.1038/ng.548.
18. Raffield, L.M., Iyengar, A.K., Wang, B., Gaynor, S.M., Spracklen, C.N., Zhong, X., Kowalski, M.H., Salimi, S., Polfus, L.M., Benjamin, E.J., et al. (2020). Allelic heterogeneity at the CRP locus identified by whole-genome sequencing in multi-ancestry cohorts. Am. J. Hum. Genet. *106*, 112–120. https://doi.org/10.1016/j.ajhg.2019.12.002.
19. Sun, Q., Graff, M., Rowland, B., Wen, J., Huang, L., Miller-Fleming, T.W., Haessler, J., Preuss, M.H., Chai, J.F., Lee, M.P., et al. (2021). Analyses of biomarker traits in diverse UK biobank participants identify associations missed by European-centric analysis strategies. J. Hum. Genet. *67*, 87–93. https://doi.org/10.1038/s10038-021-00968-0.
20. Benner, C., Spencer, C.C.A., Havulinna, A.S., Salomaa, V., Ripatti, S., and Pirinen, M. (2016). FINEMAP: efficient variable selection using summary data from genome-wide association studies. Bioinformatics (Oxford, England) *32*, 1493–1501. https://doi.org/10.1093/bioinformatics/btw018.
21. Mikhaylova, A.V., McHugh, C.P., Polfus, L.M., Raffield, L.M., Boorgula, M.P., Blackwell, T.W., Brody, J.A., Broome, J., Chami, N., Chen, M.H., et al. (2021). Whole-genome sequencing in diverse subjects identifies genetic correlates of leukocyte traits: the NHLBI TOPMed program. Am. J. Hum. Genet. *108*, 1836–1851. https://doi.org/10.1016/j.ajhg.2021.08.007.
22. Wheeler, M.M., Stilp, A.M., Rao, S., Halldórsson, B.V., Beyter, D., Wen, J., Mikhaylova, A.V., McHugh, C.P., Lane, J., Jiang, M.-Z., et al. (2021). Whole Genome sequencing identifies common and rare structural variants contributing to hematologic traits in the NHLBI TOPMed program. Preprint at medRxiv. https://doi.org/10.1101/2021.12.16.21267871.

The American Journal of Human Genetics, Volume *109*

Supplemental information

TOP-LD: A tool to explore linkage disequilibrium with TOPMed whole-genome sequence data

Le Huang, Jonathan D. Rosen, Quan Sun, Jiawen Chen, Marsha M. Wheeler, Ying Zhou, Yuan-I Min, Charles Kooperberg, Matthew P. Conomos, Adrienne M. Stilp, Stephen S. Rich, Jerome I. Rotter, Ani Manichaikul, Ruth J.F. Loos, Eimear E. Kenny, Thomas W. Blackwell, Albert V. Smith, Goo Jun, Fritz J. Sedlazeck, Ginger Metcalf, Eric Boerwinkle, NHLBI Trans-Omics for Precision Medicine (TOPMed) Consortium, Laura M. Raffield, Alex P. Reiner, Paul L. Auer, and Yun Li

Supplementary Figures

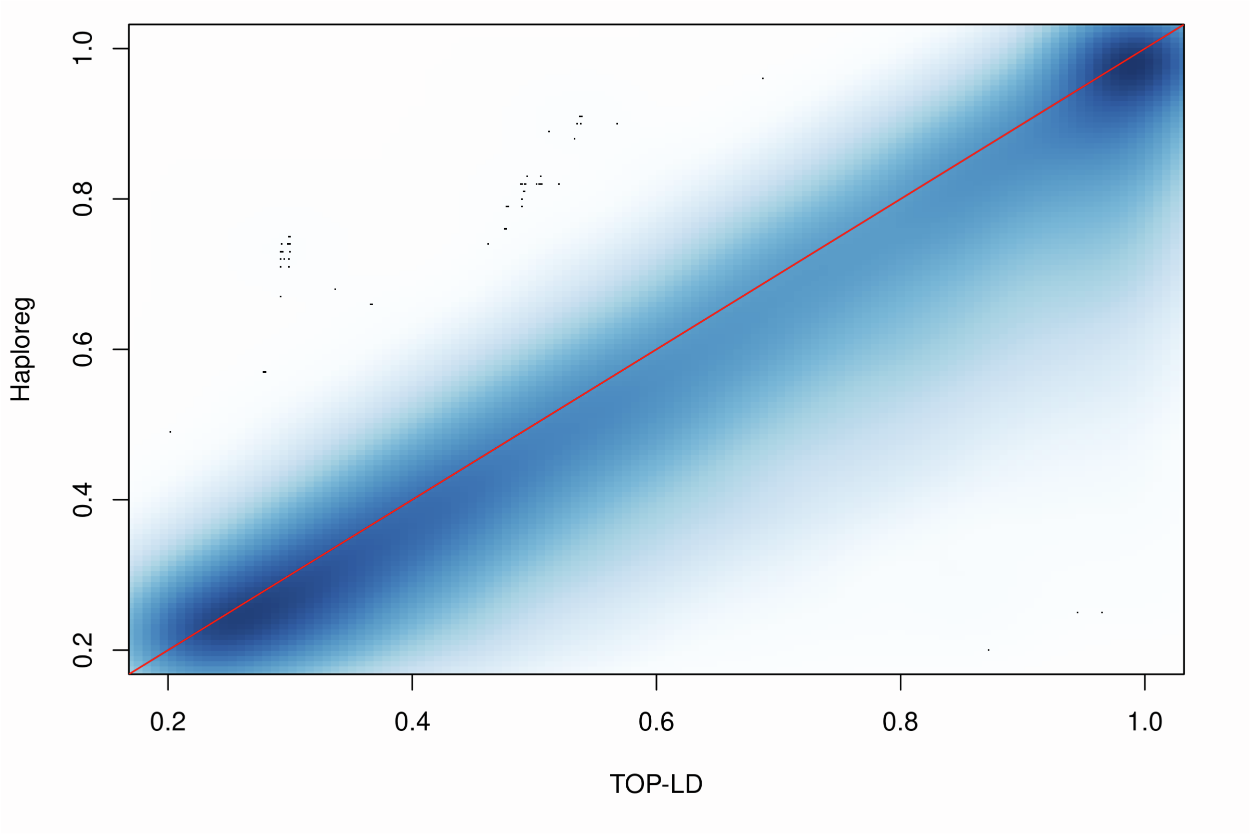

Figure S1: Smooth scatter plot of LD R-squared values from TOP-LD (x-axis) and Haploreg (y-axis) for pairs of variants with MAF>5% on chromosome 1 in European populations.

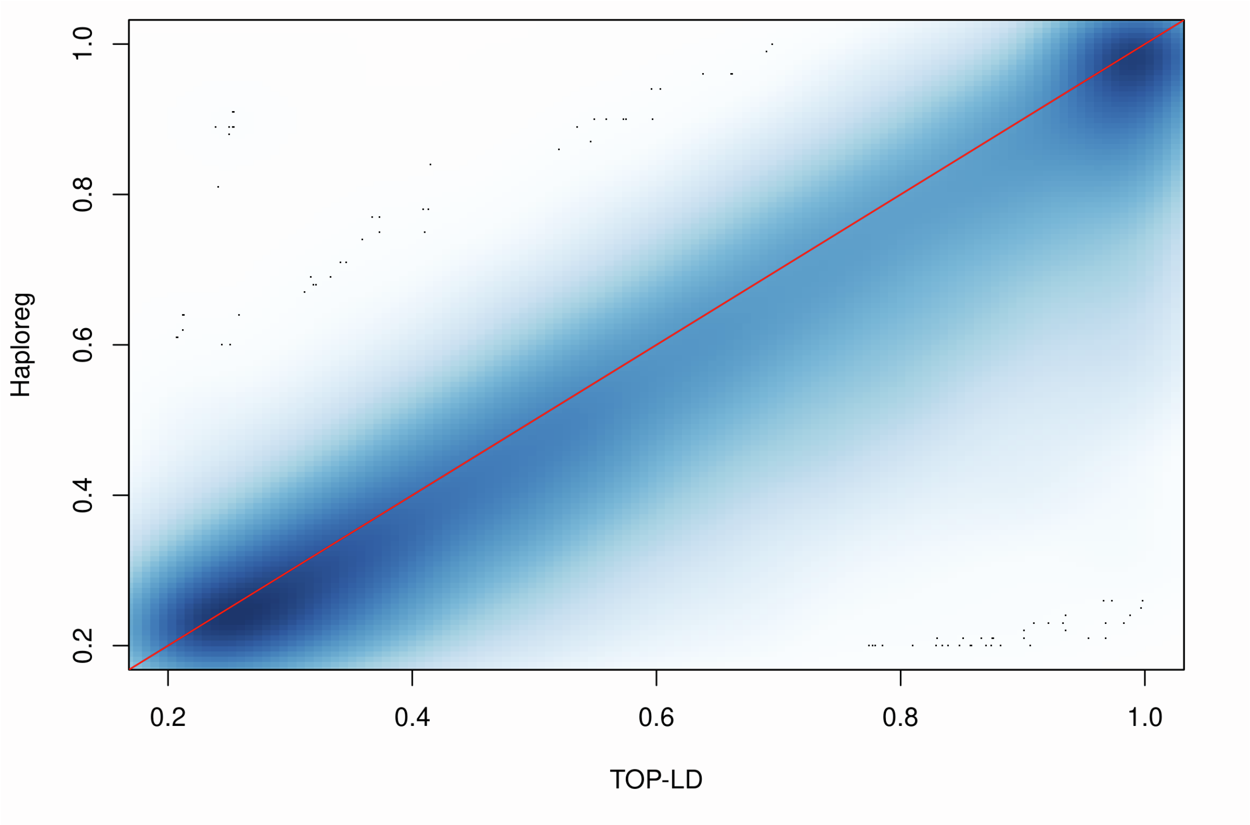

Figure S2: Smooth scatter plot of LD R-squared values from TOP-LD (x-axis) and Haploreg (y-axis) for pairs of variants with MAF>5% on chromosome 1 in African populations.

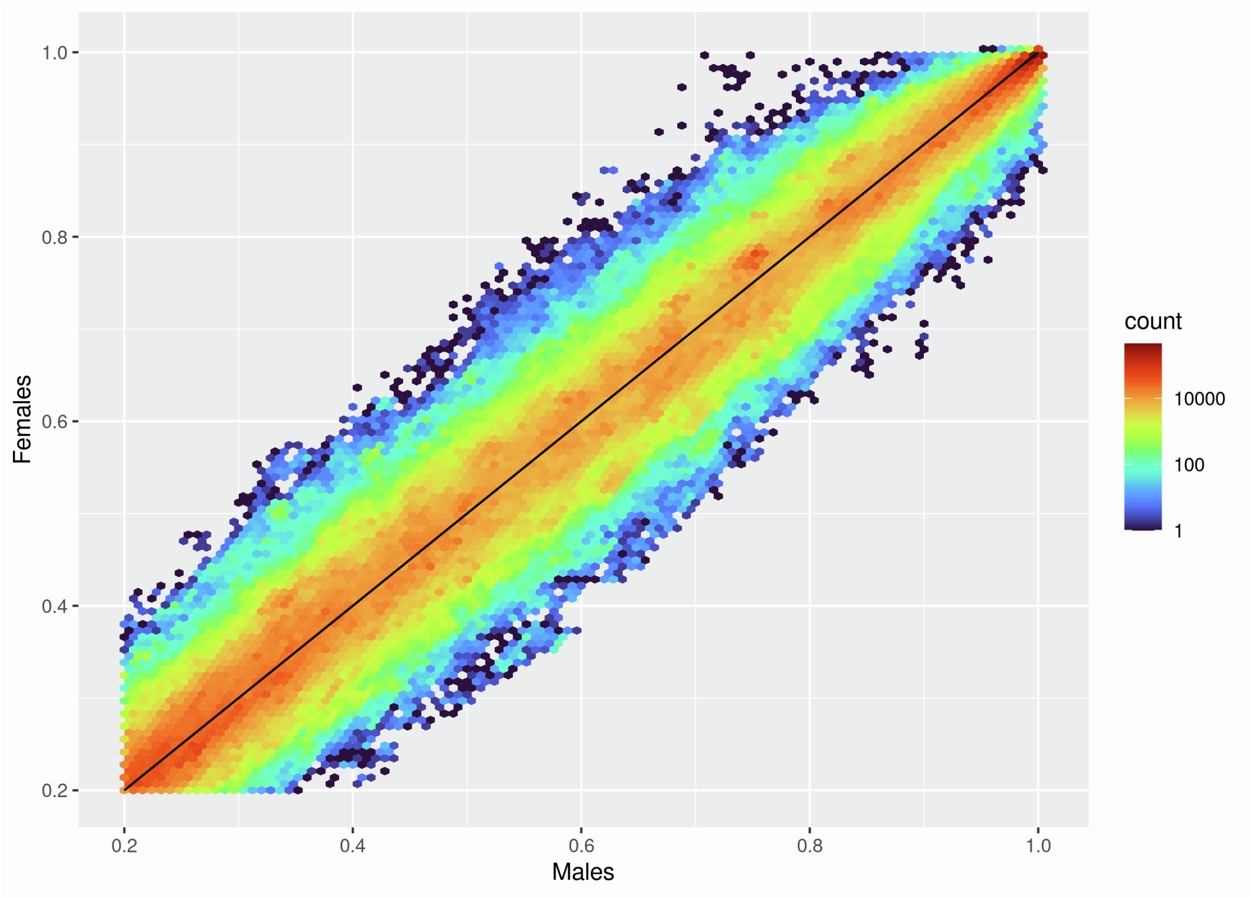

Figure S3: Hexbin plot of LD R-squared values between males (x-axis) and females (y-axis) between pairs of variants with MAF>5%, not in PAR1 or PAR2, on chromosome X in European populations.

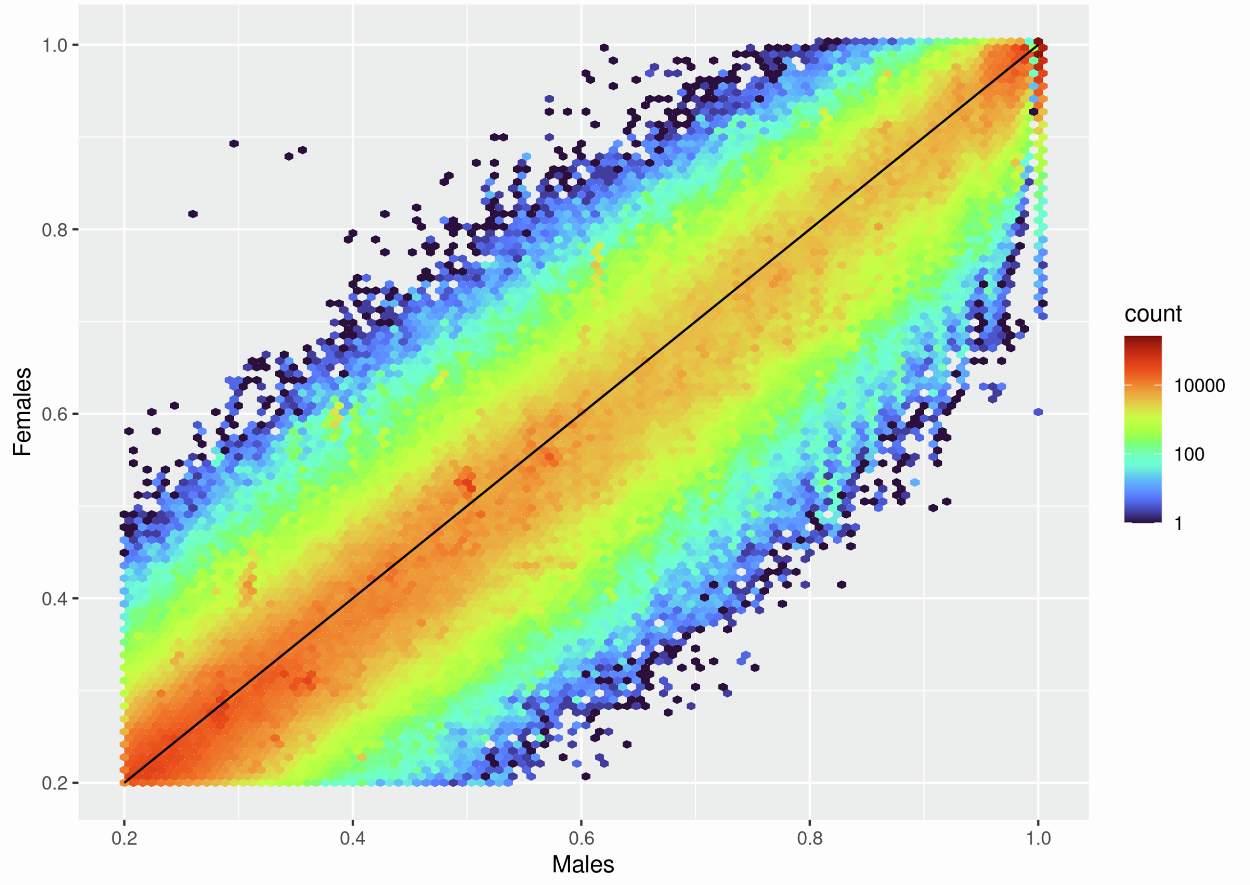

Figure S4: Hexbin plot of LD R-squared values between males (x-axis) and females (y-axis) between pairs of variants with MAF>5%, not in PAR1 or PAR2, on chromosome X in African populations.

Supplementary Tables

Table S1: Summary of SNVs and small indels by population by MAF.

Population	#TOP-LD variants[a] (MAF >0) in millions (chrX[b])	#TOP-LD variants[a] (MAF <1%) in millions (chrX[b])	#autosomal[c] variants in HaploReg4.0 in millions
EUR	153.0 (6.5)	144.0 (6.2)	16.1
AFR	62.2 (2.4)	46.2 (1.8)	25.4
SAS	23.0 (0.8)	13.3 (0.5)	13.7[d]
EAS	36.7 (1.3)	28.6 (1.1)	

a: number of unique variants, genome-wide (including autosomes and chromosome X)
b: number of unique variants on chromosome X
c: based on HaploReg LD information downloaded from https://pubs.broadinstitute.org/mammals/haploreg/data/, which does not contain chromosome X.
d: HaploReg4.0 provides LD for ASN (Asian), with no separate information for SAS and EAS.

Table S2. Summary of SNVs and small indels by population by varying LD R^2 thresholds

Population	#variants[a] ($R^2 \geq 0.2$), in millions (chrX[b])	#variants[a] ($R^2 \geq 0.5$), in millions (chrX[b])	#variants[a] ($R^2 \geq 0.8$) in millions (chrX[b])
EUR	149.8 (6.3)	141.2 (5.9)	120.2 (5.1)
AFR	62.2 (2.4)	60.0 (2.3)	53.7 (2.1)
SAS	23.0 (0.8)	21.7 (0.7)	20.4 (0.7)
EAS	36.6 (1.3)	35.0 (1.3)	31.8 (1.2)

a: number of unique variants, genome-wide (including autosomes and chromosome X) from LD pairs with $R^2 \geq$ a certain threshold
b: number of unique variants on chromosome X

Supplementary Methods

TOPMed samples

NHLBI's TOPMed program is comprised of many parent studies, including four ancestrally diverse studies that contributed to our analyses including BioMe Biobank (BioMe)[1], Jackson Heart Study (JHS)[2, 3], Multi-Ethnic Study of Atherosclerosis (MESA)[4], and Women's Health Initiative (WHI) [5]. Additional information about the design of each study and the sampling of individuals within each cohort for WGS is available in the *Cohort Descriptions* section below. All studies were approved by the appropriate institutional review boards (IRBs), and informed consent was obtained from all participants.

TOPMed whole genome sequencing and quality control

WGS was performed at an average depth of 38X by six sequencing centers (Broad Genomics, Northwest Genome Institute, Illumina, New York Genome Center, Baylor, and McDonnell Genome Institute) using Illumina X10 technology and DNA from blood. Here we report analyses from the 'Freeze 8' dataset where reads were aligned to human-genome build GRCh38 using a common pipeline across all sequencing centers. To perform variant quality control (QC) within the 'Freeze 8' dataset, a support vector machine (SVM) classifier was trained on known variant sites (positive labels) and Mendelian inconsistent variants (negative labels). Further variant filtering was done for variants with excess heterozygosity and Mendelian discordance. Sample QC measures included: concordance between annotated and inferred genetic sex, concordance between prior array genotype data and TOPMed WGS data, and pedigree checks. Details regarding the genotype 'freezes,' laboratory methods, data processing, and quality control are described on the TOPMed website and in a common document accompanying each study's dbGaP accession.

TOPMed structural variant calling and quality control

TOPMed structural variation (SV) callset release 1 was generated by Parliament2-muCNV pipeline across 138,134 multi-ethnic TOPMed WGS samples. The sample list overlaps largely with 'Freeze 8' callset except for the samples removed due to SV specific quality control issues. Parliament2 [6] is a multi-tool SV discovery pipeline that employs SV callers that have strengths in different SV types and sizes to maximize the detection sensitivity and accuracy. SVs detected by individual tools are then merged first across the callers and then across the samples using SURVIVOR [7] to generate a 'discovery' SV callset. The 'discovery' set is then genotyped and filtered by muCNV, a multi-sample SV genotyping software that performs joint genotyping based on multi-sample statistics across >100,000 samples [8]. Joint genotyping removes false discoveries by evaluating cluster separations using multi-sample distribution of read pair, split read, soft clips, and GC-corrected sequencing depth distributions. Parliament2, SURVIVOR, and muCNV are available for public access on GitHub:
https://github.com/slzarate/parliament2
https://github.com/fritzsedlazeck/SURVIVOR

https://github.com/gjun/muCNV

Analysis of Admixture

For RFMix inference, we combined samples with Native American ancestry in the Human Genome Diversity Project (HGDP) [9] and samples with African, East Asian, European and South Asian ancestries in the 1000 Genomes Project (1000G) [10]. We first retained variants that are available both in HGDP and in 1000G, then performed LD pruning using PLINK [11] with R^2 threshold of 0.01. ADMIXTURE [12] global ancestry analysis for HGDP samples identified 92 Native American samples with ≥90% Native American ancestry. To attain balanced sample size recommended for RFMix inference, we randomly selected 92 samples from each ancestry in the 1000G dataset.

Cohort Descriptions

BioMe

The Charles Bronfman Institute for Personalized Medicine at Mount Sinai Medical Center (MSMC), BioMe Biobank, founded in September 2007, is an ongoing, broadly-consented electronic health record-linked clinical care biobank that enrolls participants non-selectively from the Mount Sinai Medical Center patient population. The MSMC serves diverse local communities of upper Manhattan, including Central Harlem (86% African American), East Harlem (88% Hispanic/Latino), and Upper East Side (88% Caucasian/White) with broad health disparities.

JHS

The Jackson Heart Study (JHS, https://www.jacksonheartstudy.org/jhsinfo/) is a large, community-based, observational study whose participants were recruited from urban and rural areas of the three counties (Hinds, Madison and Rankin) that make up the Jackson, MS metropolitan statistical area (MSA). Participants were enrolled from each of 4 recruitment pools: random, 17%; volunteer, 30%; currently enrolled in the Atherosclerosis Risk in Communities (ARIC) Study, 31% and secondary family members, 22%. Recruitment was limited to non-institutionalized adult African Americans 35-84 years old, except in a nested family cohort where those 21 to 34 years of age were also eligible. The final cohort of 5,306 participants included 6.59% of all African American Jackson MSA residents aged 35-84 during the baseline exam (N-76,426, US Census 2000). Among these, approximately 3,700 gave consent that allows genetic research and deposition of data into dbGaP. Major components of three clinic examinations (Exam 1 – 2000-2004; Exam 2 – 2005-2008; Exam 3 – 2009-2013) include medical history, physical examination, blood/urine analytes and interview questions on areas such as: physical activity; stress, coping and spirituality; racism and discrimination; socioeconomic position; and access to health care. A fourth exam is ongoing. Extensive clinical phenotyping includes anthropometrics, electrocardiography, carotid ultrasound, ankle-brachial blood pressure index, echocardiography, CT chest and abdomen for coronary and aortic calcification, liver fat, and

subcutaneous and visceral fat measurement, and cardiac MRI. At 12-month intervals after the baseline clinic visit (Exam 1), participants have been contacted by telephone to: update information; confirm vital statistics; document interim medical events, hospitalizations, and functional status; and obtain additional sociocultural information. Questions about medical events, symptoms of cardiovascular disease and functional status are repeated annually. Ongoing cohort surveillance includes abstraction of medical records and death certificates for relevant International Classification of Diseases (ICD) codes and adjudication of nonfatal events and deaths. CMS data are currently being incorporated into the dataset.

MESA

The MESA study is a study of the characteristics of subclinical cardiovascular disease (disease detected non-invasively before it has produced clinical signs and symptoms) and the risk factors that predict progression to clinically overt cardiovascular disease or progression of subclinical disease. MESA researchers study a diverse, population-based sample of 6,814 asymptomatic men and women aged 45-84. Thirty-eight percent of the recruited participants are white, 28 percent African-American, 22 percent Hispanic, and 12 percent Asian, predominantly of Chinese descent. Participants were recruited from six field centers across the United States: Wake Forest University, Columbia University, Johns Hopkins University, University of Minnesota, Northwestern University and the University of California - Los Angeles.

WHI

The Women's Health Initiative (WHI) is a long-term, prospective, multi-center cohort study that investigates post-menopausal women's health 8. WHI was funded by the National Institutes of Health and the National Heart, Lung, and Blood Institute to study strategies to prevent heart disease, breast cancer, colon cancer, and osteoporotic fractures in women 50-79 years of age. WHI involves 161,808 women recruited between 1993 and 1998 at 40 centers across the US. The study consists of two parts: the WHI Clinical Trial which was a randomized clinical trial of hormone therapy, dietary modification, and calcium/Vitamin D supplementation, and the WHI Observational Study, which focused on many of the inequities in women's health research and provided practical information about the incidence, risk factors, and interventions related to heart disease, cancer, and osteoporotic fractures.

Acknowledgements

TOPMed Accession #	TOPMed Project	Parent Study Name	TOPMed Phase	Omics Center	Omics Support
phs001644	BioMe	BioMe	3	Baylor	HHSN268201600033I
phs001644	BioMe	BioMe	3	MGI	HHSN268201600037I
phs000964	JHS	JHS	1	NWGC	HHSN268201100037C

phs001416	AA_CAC	MESA AA_CAC	2	Broad Genomics	HHSN268201500014C
phs001416	MESA	MESA	2	Broad Genomics	3U54HG003067-13S1
phs001237	WHI	WHI	2	Broad Genomics	HHSN268201500014C

BioMe: The Mount Sinai BioMe Biobank has been supported by The Andrea and Charles Bronfman Philanthropies and in part by Federal funds from the NHLBI and NHGRI (U01HG00638001; U01HG007417; X01HL134588). We thank all participants in the Mount Sinai Biobank. We also thank all our recruiters who have assisted and continue to assist in data collection and management and are grateful for the computational resources and staff expertise provided by Scientific Computing at the Icahn School of Medicine at Mount Sinai.

JHS: The Jackson Heart Study (JHS) is supported and conducted in collaboration with Jackson State University (HHSN268201800013I), Tougaloo College (HHSN268201800014I), the Mississippi State Department of Health (HHSN268201800015I) and the University of Mississippi Medical Center (HHSN268201800010I, HHSN268201800011I and HHSN268201800012I) contracts from the National Heart, Lung, and Blood Institute (NHLBI) and the National Institute on Minority Health and Health Disparities (NIMHD). The authors also wish to thank the staffs and participants of the JHS.

The views expressed in this manuscript are those of the authors and do not necessarily represent the views of the National Heart, Lung, and Blood Institute; the National Institutes of Health; or the U.S. Department of Health and Human Services.

MESA: MESA and the MESA SHARe project are conducted and supported by the National Heart, Lung, and Blood Institute (NHLBI) in collaboration with MESA investigators. Support for MESA is provided by contracts 75N92020D00001, HHSN268201500003I, N01-HC-95159, 75N92020D00005, N01-HC-95160, 75N92020D00002, N01-HC-95161, 75N92020D00003, N01-HC-95162, 75N92020D00006, N01-HC-95163, 75N92020D00004, N01-HC-95164, 75N92020D00007, N01-HC-95165, N01-HC-95166, N01-HC-95167, N01-HC-95168, N01-HC-95169, UL1-TR-000040, UL1-TR-001079, UL1-TR-001420. MESA Family is conducted and supported by the National Heart, Lung, and Blood Institute (NHLBI) in collaboration with MESA investigators. Support is provided by grants and contracts R01HL071051, R01HL071205, R01HL071250, R01HL071251, R01HL071258, R01HL071259, by the National Center for Research Resources, Grant UL1RR033176. The provision of genotyping data was supported in part by the National Center for Advancing Translational Sciences, CTSI grant UL1TR001881, and the National Institute of Diabetes and Digestive and Kidney Disease Diabetes Research Center (DRC) grant DK063491 to the Southern California Diabetes Endocrinology Research Center.

WHI: The WHI program is funded by the National Heart, Lung, and Blood Institute, National Institutes of Health, U.S. Department of Health and Human Services through contracts

HHSN268201600018C, HHSN268201600001C, HHSN268201600002C, HHSN268201600003C, and HHSN268201600004C.

The views expressed in this manuscript are those of the authors and do not necessarily represent the views of the National Heart, Lung, and Blood Institute; the National Institutes of Health; or the U.S. Department of Health and Human Services.

References

1. Gottesman, O., et al., *The Electronic Medical Records and Genomics (eMERGE) Network: past, present, and future.* Genet Med, 2013. **15**(10): p. 761-71.
2. Taylor, H.A., Jr., et al., *Toward resolution of cardiovascular health disparities in African Americans: design and methods of the Jackson Heart Study.* Ethn Dis, 2005. **15**(4 Suppl 6): p. S6-4-17.
3. Wilson, J.G., et al., *Study design for genetic analysis in the Jackson Heart Study.* Ethn Dis, 2005. **15**(4 Suppl 6): p. S6-30-37.
4. Bild, D.E., et al., *Multi-Ethnic Study of Atherosclerosis: objectives and design.* Am J Epidemiol, 2002. **156**(9): p. 871-81.
5. *Design of the Women's Health Initiative clinical trial and observational study. The Women's Health Initiative Study Group.* Control Clin Trials, 1998. **19**(1): p. 61-109.
6. Zarate, S., et al., *Parliament2: Accurate structural variant calling at scale.* Gigascience, 2020. **9**(12).
7. Jeffares, D.C., et al., *Transient structural variations have strong effects on quantitative traits and reproductive isolation in fission yeast.* Nat Commun, 2017. **8**: p. 14061.
8. Jun, G., et al., *muCNV: Genotyping Structural Variants for Population-level Sequencing.* Bioinformatics, 2021.
9. Li, J.Z., et al., *Worldwide human relationships inferred from genome-wide patterns of variation.* Science, 2008. **319**(5866): p. 1100-4.
10. Genomes Project, C., et al., *A global reference for human genetic variation.* Nature, 2015. **526**(7571): p. 68-74.
11. Chang, C.C., et al., *Second-generation PLINK: rising to the challenge of larger and richer datasets.* Gigascience, 2015. **4**: p. 7.
12. Alexander, D.H., J. Novembre, and K. Lange, *Fast model-based estimation of ancestry in unrelated individuals.* Genome Res, 2009. **19**(9): p. 1655-64.
